# Supplementary material for: Redox activation of ATM enhances GSNOR translation to sustain mitophagy and tolerance to oxidative stress
Source: EMBO Rep. 2020 Nov 27;22(1):e50500. doi: 10.15252/embr.202050500 (PMC7788447; doi:10.15252/embr.202050500)
Supplement: Supplementary file 1 — Appendix [file EMBR-22-e50500-s001.docx]

**Redox activation of ATM enhances GSNOR translation to sustain mitophagy and tolerance to oxidative stress**

**APPENDIX FIGURES**

**Table of content:**

Appendix Figure S1. Complementary data for Figure 4 2

Appendix Figure S2. U2OS cells stably expressing doxycycline-inducible alleles coding for the *wild-type* (ATMWT), the DNA-damage insensitive (R2579A/R2580A, ATM2RA) or the redox insensitive (C2991L, ATMCL) forms of ATM. 3

Appendix Figure S3. Analysis of dead cells. 4

Appendix Figure S4. Efficiency of transfection. 5

Appendix Figure S5. CD4^+^ T cell purification. 6

Appendix Figure S6. Pharmacological inhibitors of Nrf2, ATM, p53 and Chk2 do not alter GSNOR levels *per se*. 7

Appendix Figure S7. Fluorescence microscopy analysis of mitophagy. 8

Appendix Figure S1. Complementary data for Figure 4

**
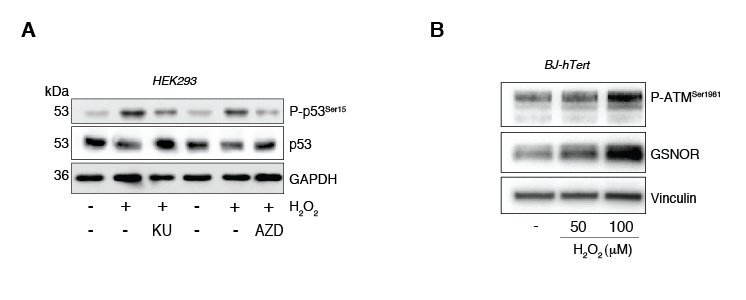
**

**A.** Western blot analysis of the basal and phospho-active forms of p53 in HEK293 cells treated for 24 h with 100 μM H_2_O_2_ in the presence or absence of the ATM inhibitor KU55933 (*KU*), or the CHK2 inhibitor AZD7762 (*AZD*). **B.** Western blot analysis of ATM and GSNOR in BJ-hTERT cells treated with 50 or 100 μM H2O2.

Appendix Figure S2. U2OS cells stably expressing doxycycline-inducible alleles coding for the *wild-type* (ATMWT), the DNA-damage insensitive (R2579A/R2580A, ATM2RA) or the redox insensitive (C2991L, ATMCL) forms of ATM.

**
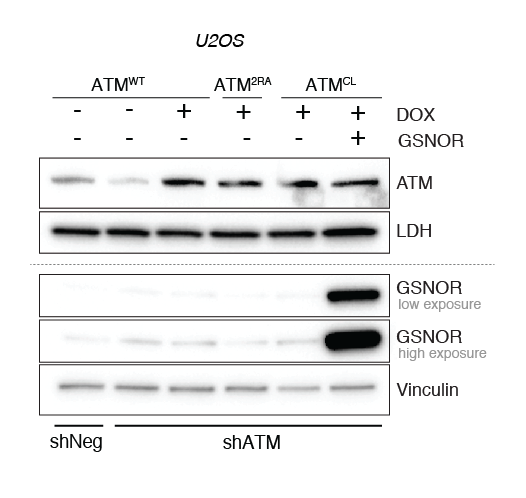
**

Western blot analysis of ATM and GSNOR in U2OS cells silenced for ATM (shATM) and treated with doxycycline to induce ATM^WT^, ATM^CL^ or ATM^2RA^ mutant expressions. LDH and Vinculin were selected as loading control.

Appendix Figure S3. Analysis of dead cells.


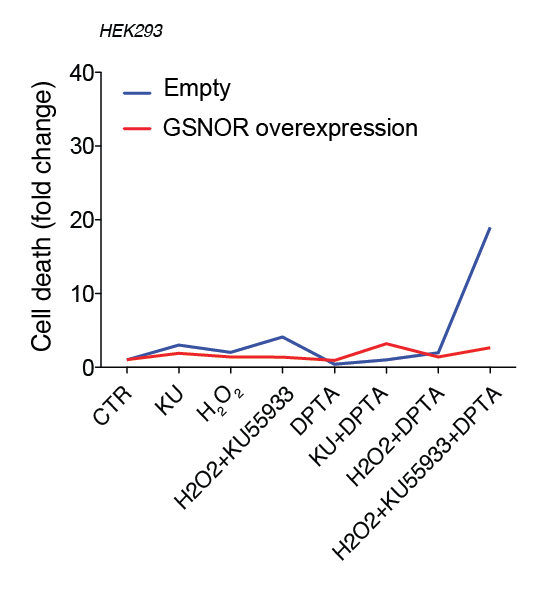


Analysis of dead cells performed with Trypan blue exclusion assay in HEK293 cells overexpressing the *wild type* form of GSNOR (GSNORWT) or an empty vector (Empty). Cells where treated with ATM inhibitor KU-55933 (*KU*), 200 μM H2O2, 400 μM DPTA, alone or in combination. Data are shown as fold change of dead cells relative to untreated cells (arbitrarily set to 1) and represent the mean count n = 4 experiments done in duplicate.

Appendix Figure S4. Efficiency of transfection.

**
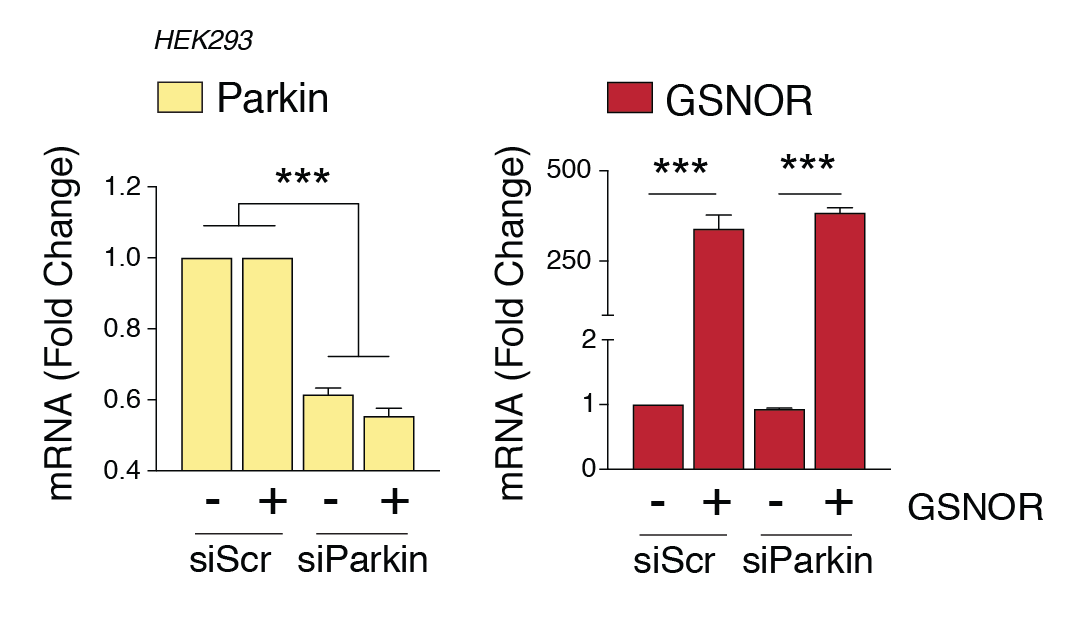
**

RT-qPCR analyses of (*left*) Parkin and (*right*) GSNOR expression in HEK293 co-transfected with siRNA against Parkin (siParkin) and a plasmid coding for GSNOR. siScr was used as control. Results shown are the means ± SD of n = 3 biological replicates. Significance was calculated by unpaired two-tailed t-test ****p* < 0.001*.*

Appendix Figure S5. CD4^+^ T cell purification.

**
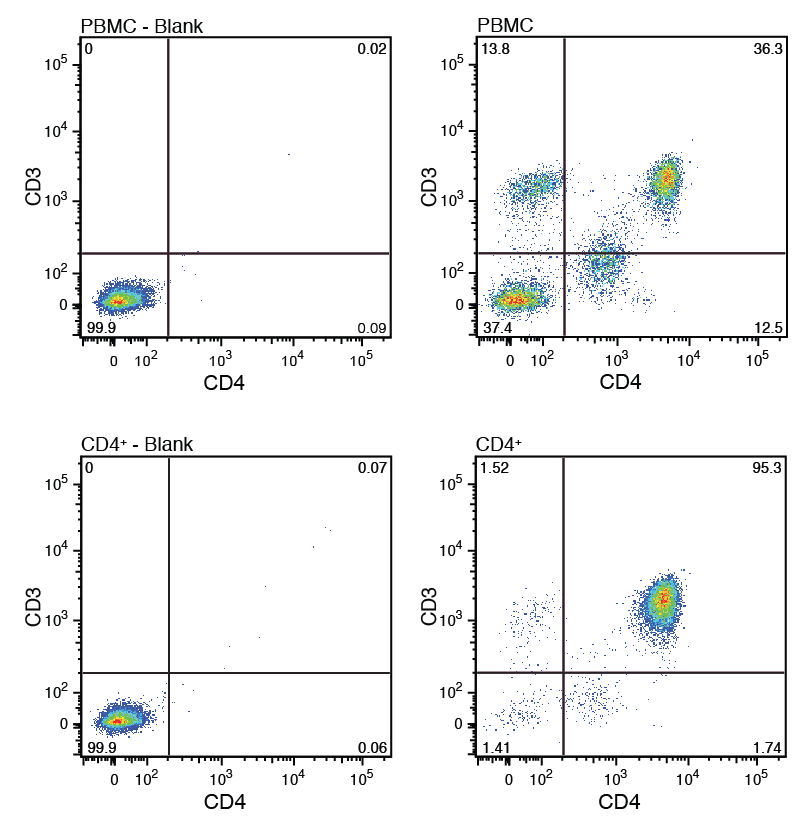
**

CD4^+^ T lymphocyte purification was assessed by flow cytometry (FACSCelesta, BD) by CD4 and CD3 staining, starting for peripheral blood mononuclear cells (PBMC) isolated by Ficoll density gradient centrifugation and sorted using “CD4^+^ T Cell Isolation Kit, human”. Purity of isolated CD4^+^ T cells was in the range of 95%.

Appendix Figure S6. Pharmacological inhibitors of Nrf2, ATM, p53 and Chk2 do not alter GSNOR levels *per se*.

**
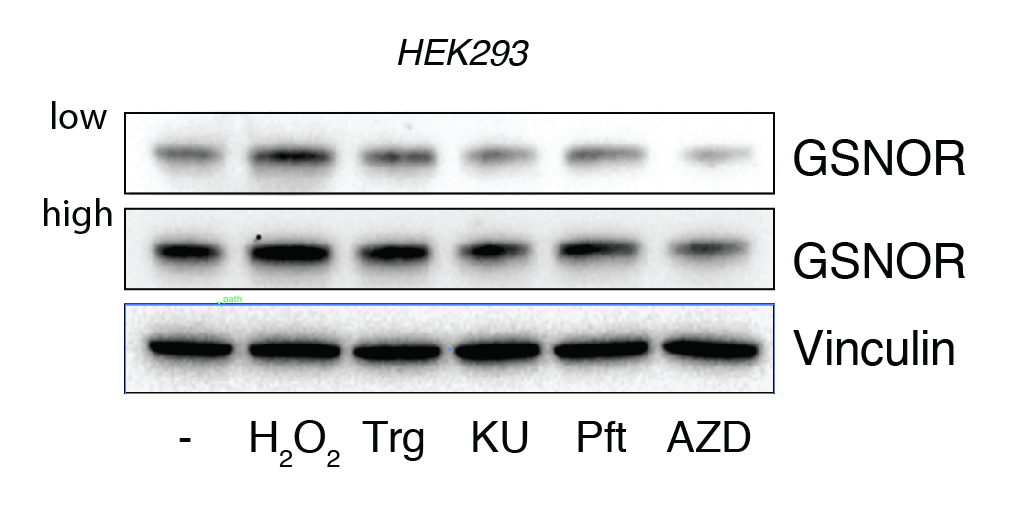
**

Western blot analysis of GSNOR in HEK293 cells treated for 24 h with 2.5 μM Trigonelline (Trg), 10 μM KU55933 (KU), 20 μM pifithrin-α (Pft), or 20 nM AZD7762 (AZD). Vinculin was selected as loading control.

Appendix Figure S7. Fluorescence microscopy analysis of mitophagy.

**
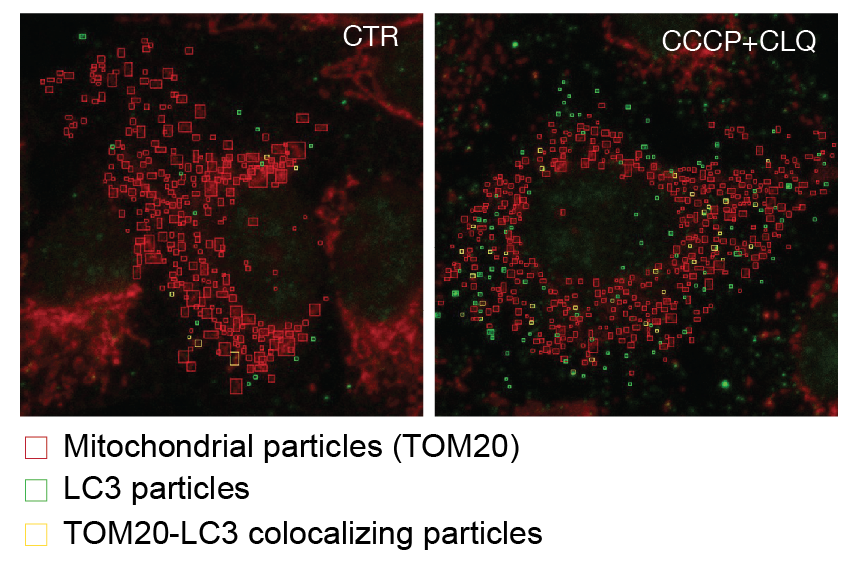
**

Model of the analysis software applied to evaluate mitophagy by calculating colocalization between green and red fluorescence.
