# Supplementary material for: Redox activation of ATM enhances GSNOR translation to sustain mitophagy and tolerance to oxidative stress
Source: EMBO Rep. 2020 Nov 27;22(1):e50500. doi: 10.15252/embr.202050500 (PMC7788447; doi:10.15252/embr.202050500)
Supplement: Supplementary file 2 — Expanded View Figures PDF [file EMBR-22-e50500-s002.pdf]

Expanded View Figures

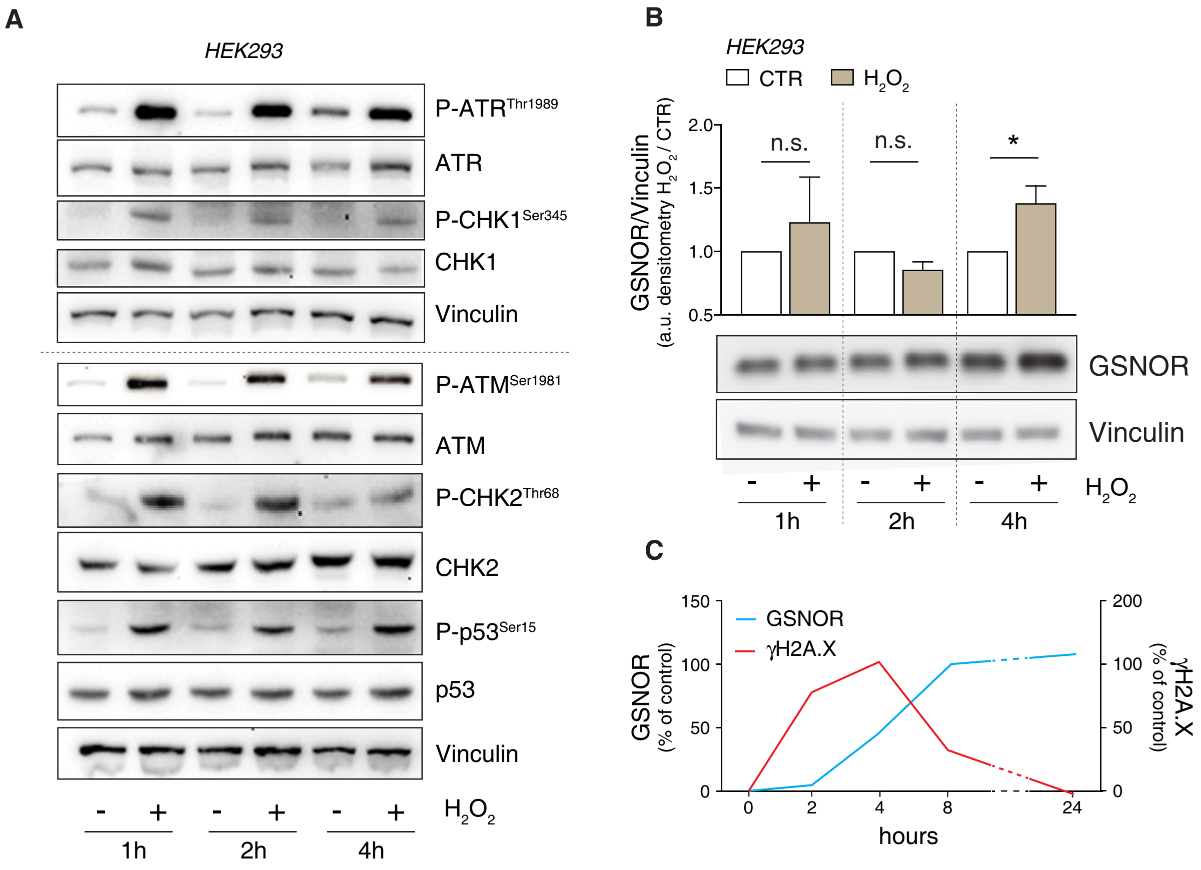

Figure EV1. H_2O_2 rapidly induces ATR/CHK1 and ATM/CHK2 signaling axes, but not GSNOR.

A HEK293 cells were treated for 1, 2 and 4 h with 100 μM H_2O_2. Vinculin was used as loading control. Basal and phospho-active forms of ATM, CHK2, ATR, CHK1, and p53 were assessed by Western blot.

B Western blot analysis performed in the same experimental setting described in panel A. Vinculin was used as loading control. Densitometry of GSNOR immunoreactive bands is normalized to Vinculin and expressed as arbitrary units. Values shown are the means ± SD of n = 3 different experiments. *$P < 0.05$; *n.s.*, not significant.

C Integrated time-dependent profiles of GSNOR (extracted from combining data of Figs EV1B and 1A) and phospho-histone H2A.X (extracted from Fig 2B) were evaluated in HEK293 cells treated with 100 μM H_2O_2. Data are expressed as % of control (untreated cells).

© 2020 The Authors

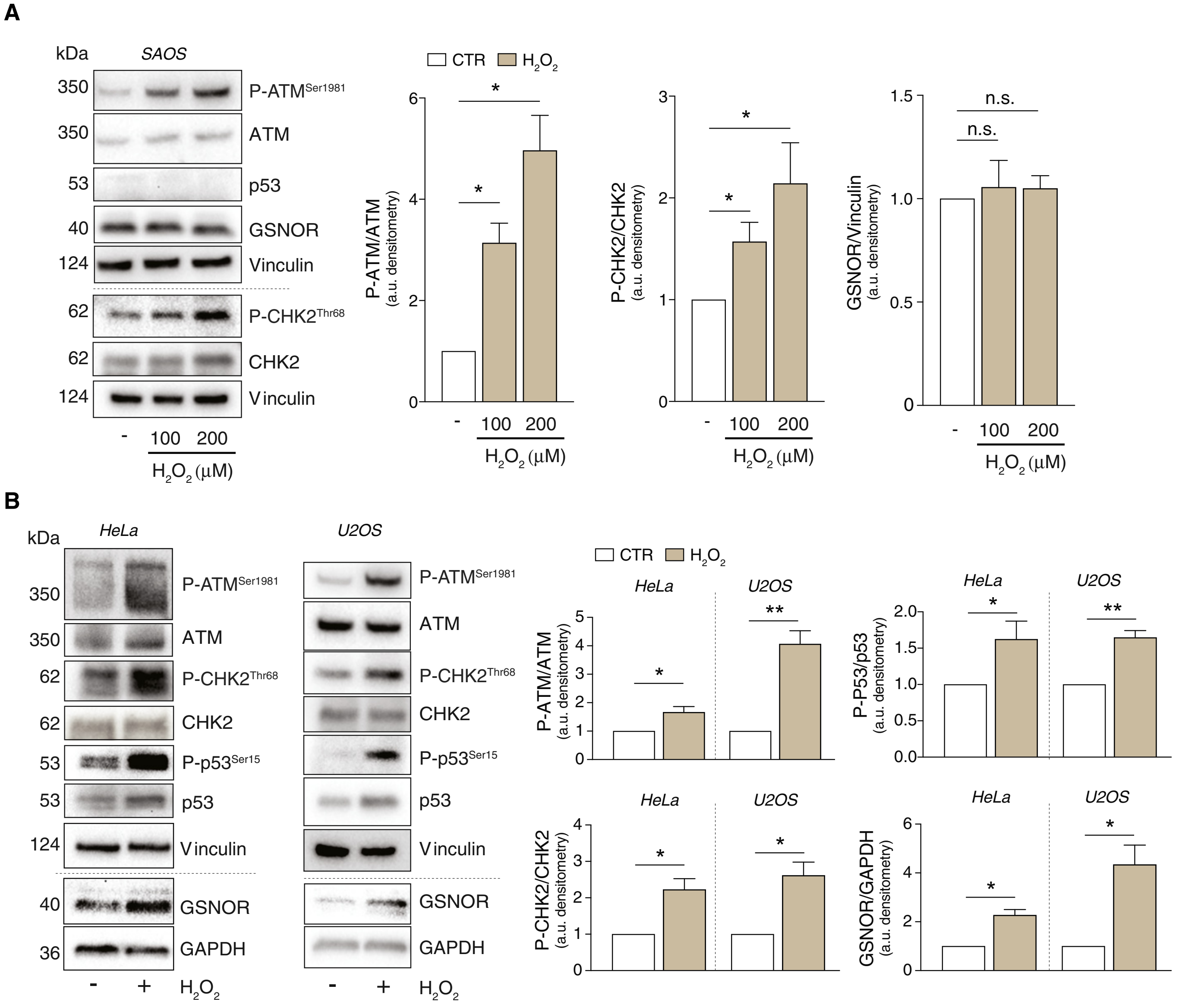

Figure EV2. GSNOR is induced by H_2O_2 in p53 expressing cells.

A, B SAOS (A), HeLa, and U2OS cells (B) were treated for 24 h with 100 or 200 µM H_2O_2. Basal and phospho-active forms of ATM, CHK2, and p53 and GSNOR were assessed by Western blot. Phospho:basal level ratios of ATM, CHK2 and p53 (normalized to Vinculin), along with densitometry of GSNOR immunoreactive bands (normalized to GAPDH), are expressed as arbitrary units. Values shown represent the means ± SD of *n* = 3 different experiments. *$P < 0.05$; **$P < 0.01$; *n.s.*, not significant.

 © 2020 The Authors

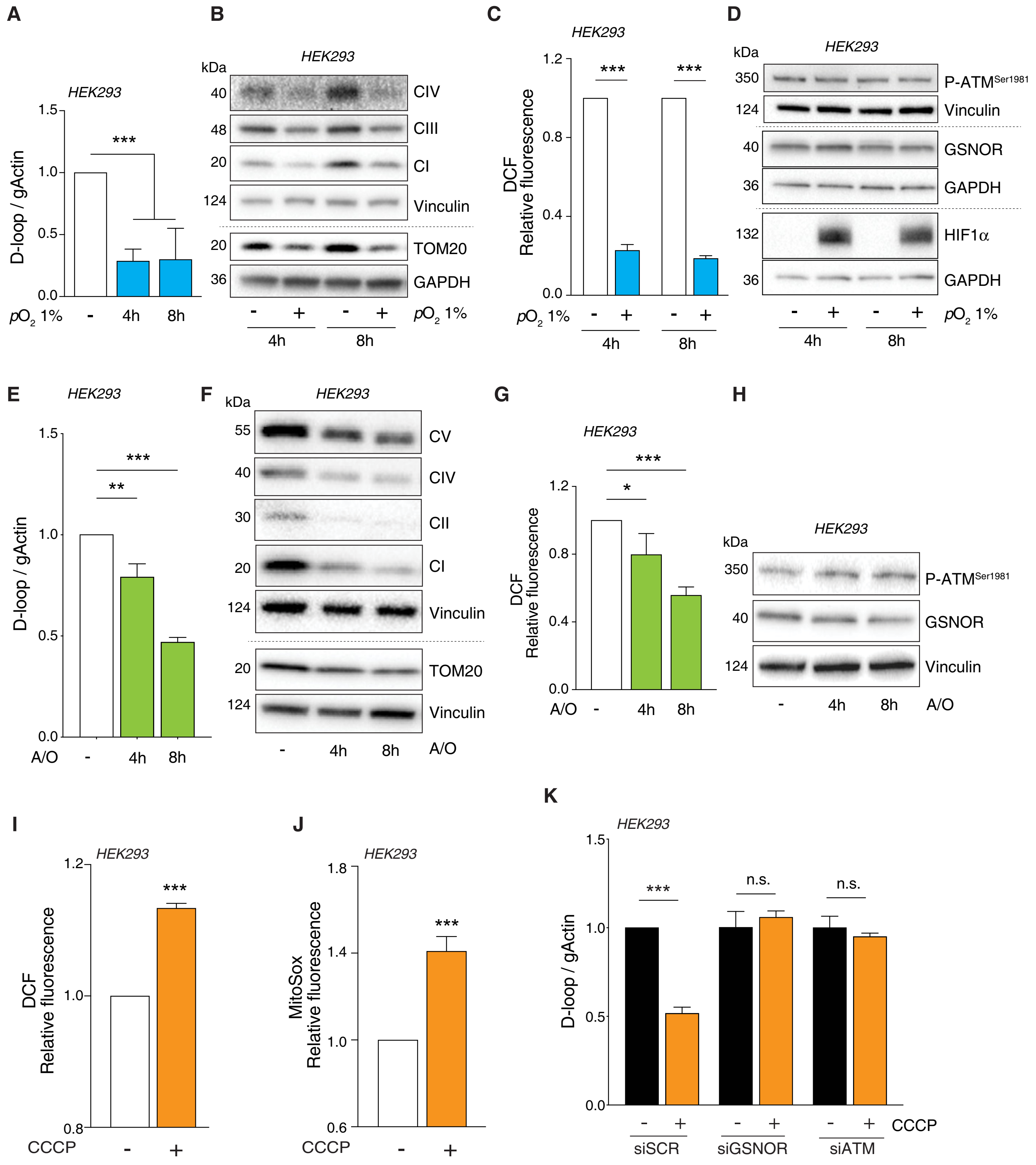
A
B
C
D
E
F
G
H
I
J
K
HEK293
D-loop / gActin
DCF
Relative fluorescence
MitoSox
kDa
CV
CIV
CIII
CII
CI
Vinculin
TOM20
GAPDH
P-ATMSer1981
GSNOR
HIF1α
pO2 1%
4h
8h
A/O
CCCP
siSCR
siGSNOR
siATM
***
**
*
n.s.

Figure EV3.

Figure EV3. Mitophagy is kept sustained by ATM/GSNOR axis as a selective response to H_2O_2.

A–D HEK293 cells were subjected to hypoxia (i.e., pO_2 = 1%) for 4 and 8 h. (A) Mitophagy was assayed by RT–qPCR relative quantitation of D-loop (selected as measure of mtDNA). Results shown are the means ± SD of n = 6 independent experiments. ***P < 0.001 calculated with respect to cells kept in normoxic conditions. (B) Mitophagy was also evaluated by Western blot of TOM20 and different mitochondrial complex subunits, i.e., NDUFB8 (CI), SDHB (CII), and MTCO2 (complex IV). Vinculin and GAPDH were used as loading controls. (C) After treatment, cells were incubated with 2′,7′-H_2DCF-DA to cytofluorometrically assess the intracellular production of H_2O_2. Values are shown as units of DCF fluorescence relative to cells maintained in normoxic conditions (arbitrarily set as 1) and represent the means ± SD of n = 3 independent experiments. ***P < 0.001. (D) Western blot analysis of phospho-ATM and GSNOR. HIF1α was selected as marker of hypoxia. Vinculin and GAPDH were used as loading controls.

E–H HEK293 cells were treated for 4 and 8 h with 1 µM of a combination of oligomycin and antimycin. Mitophagy was evaluated by (E) D-loop quantitation as described in panel A. Results shown represent the means ± SD of n = 3 independent experiments. **P < 0.01 and ***P < 0.001 with respect to untreated cells. (F) Western blot of different mitochondrial proteins (as in panel B). Vinculin was used as loading control. (G) H_2O_2 production was evaluated cytofluorometrically as described in panel C. Results shown represent the means ± SD of n = 3 independent experiments. *P < 0.05; ***P < 0.01 with respect to untreated cells. (H) Western blot analysis of phospho-ATM and GSNOR. Vinculin and GAPDH was used as loading control.

I–K HEK293 cells were treated for 8 h with 10 µM CCCP. After treatment, cells were incubated with 5 µM 2′,7′-H_2DCF-DA (I) or MitoSox (J) to evaluate the production of H_2O_2 or mitochondrial superoxide, respectively. Values are shown as units of DCF or MitoSox fluorescence relative to untreated cells (arbitrarily set as 1) and represent the means ± SEM (I) or SD (J) of n = 3 independent experiments. ***P < 0.001 with respect to untreated cells. (K) Before CCCP treatment, cells were transfected for 48 h with siRNA against ATM (siATM), GSNOR (siGSNOR), or control siRNA (*scramble*, siScr). Mitophagy was assessed by RT–qPCR relative quantitation of D-loop. Results shown are the means ± SD of n = 3 experiments run in triplicate. ***P < 0.001; *n.s.*, not significant, calculated with regard to untreated cells.

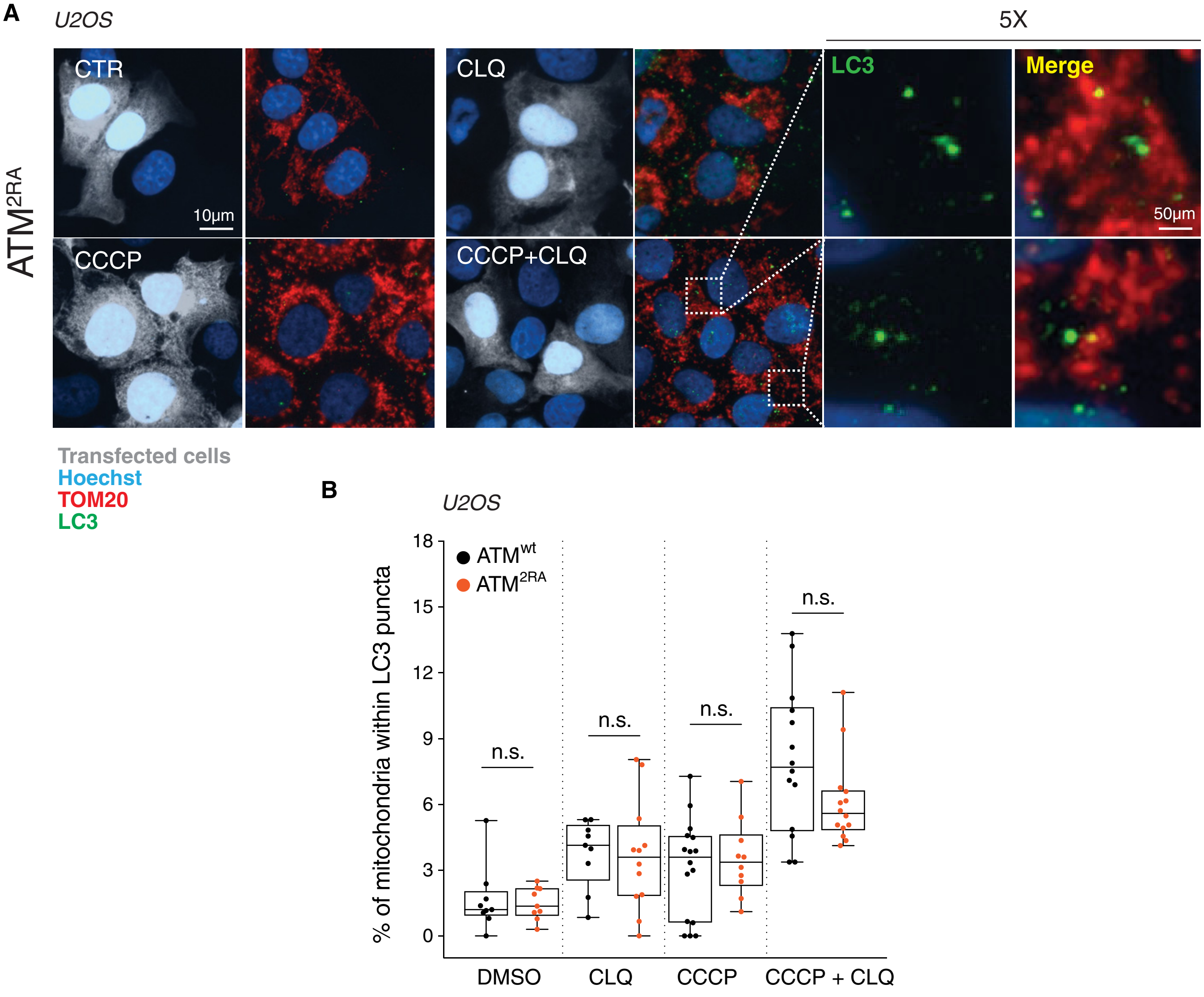

Figure EV4.

 © 2020 The Authors

◀ **Figure EV4. The DNA damage-unresponsive ATM mutant (ATM^{2RA}) does not affect mitophagy induced by CCCP.**

A ATM^{2RA} U2OS cells were treated for 8 h with 1 μM CCCP and incubated with chloroquine (CLQ) to enhance differences in mitophagy. Anti-TOM20 (red) was used to visualize mitochondria; anti-LC3 (green) was used to identify autophagosomes.

B Percentage of mitochondria merging with LC3-positive puncta calculated by Fiji analysis software using the open-source plugin ComDet v. 0.3.7. Values are expressed as % of mitochondria ($TOM20^+$ particles) co-localizing with LC3/cell and graphed as boxes (25th–75th interquartile range) and whiskers (minimum to maximum showing all points), with central bands representing the median of $n \geq 9$ different cells. *n.s.*, not significant.

© 2020 The Authors
