# Supplementary figures and images for: Redox activation of ATM enhances GSNOR translation to sustain mitophagy and tolerance to oxidative stress
Source: EMBO Rep. 2020 Nov 27;22(1):e50500. doi: 10.15252/embr.202050500 (PMC7788447; doi:10.15252/embr.202050500)

**FIG EV1A**

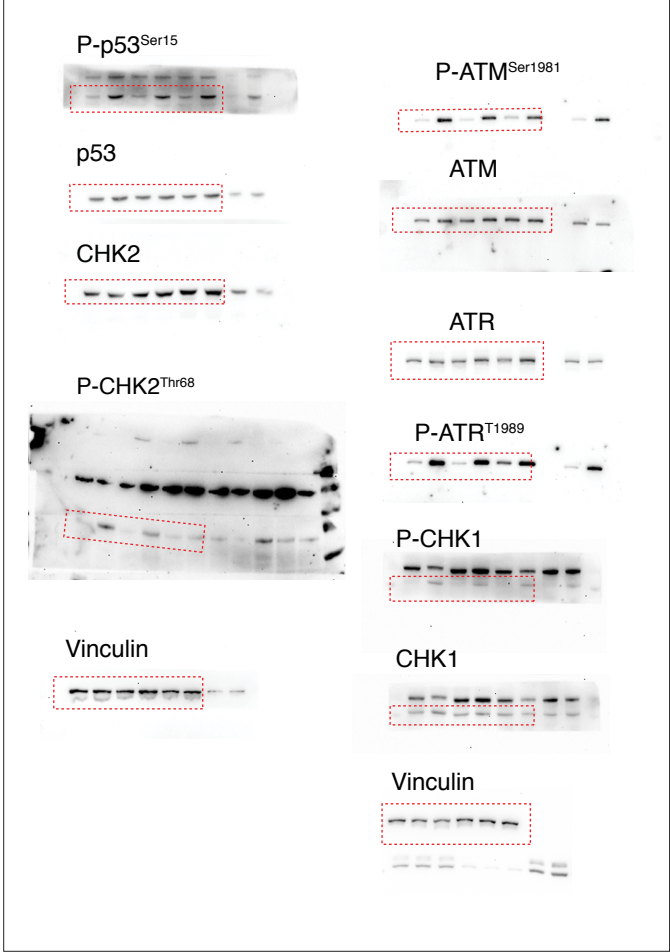

**FIG EV1B**

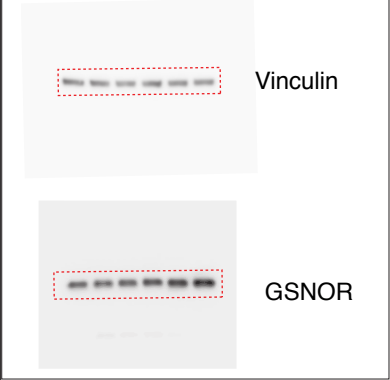

Supplement: Supplementary file 3 — Source Data for Expanded View [file EMBR-22-e50500-s011.zip › EV_Figure_Source_Data/EMBOR-2020-50500V3-FigureEV1_Source_Data-sd/FigureEV1_uncropped gels.pdf]

**FIG EV3B**

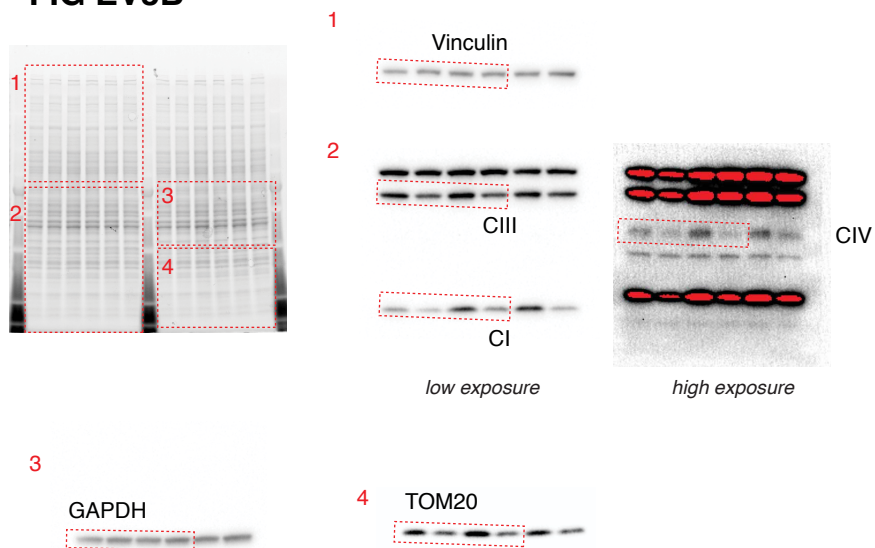

**FIG EV3D**

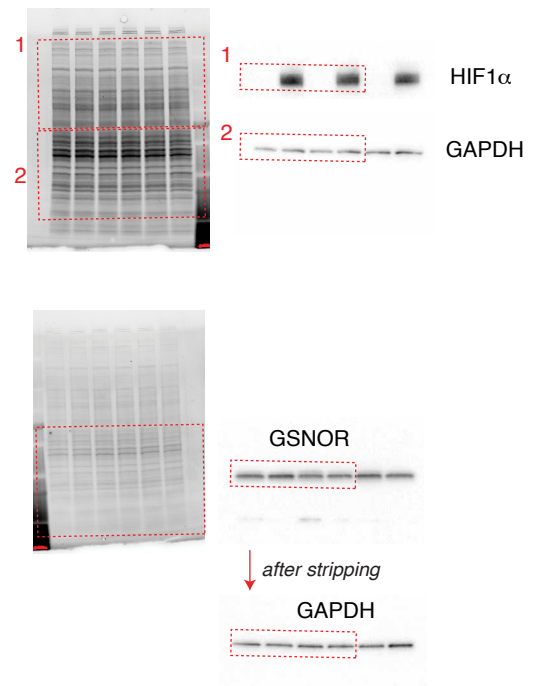

**FIG EV3H**

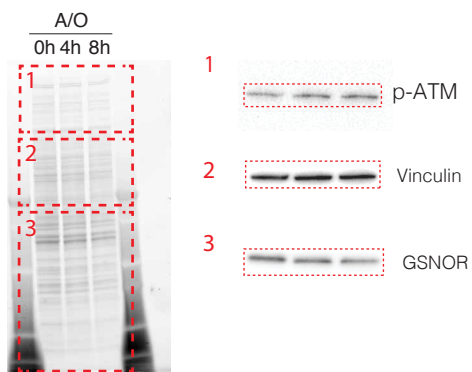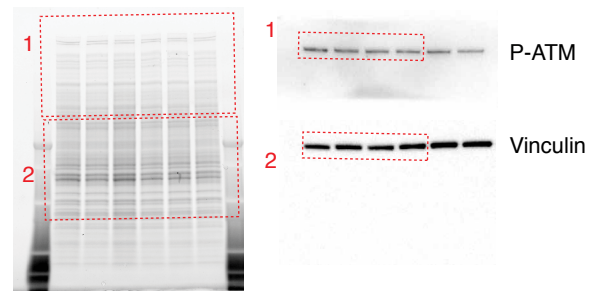

**FIG EV3F**

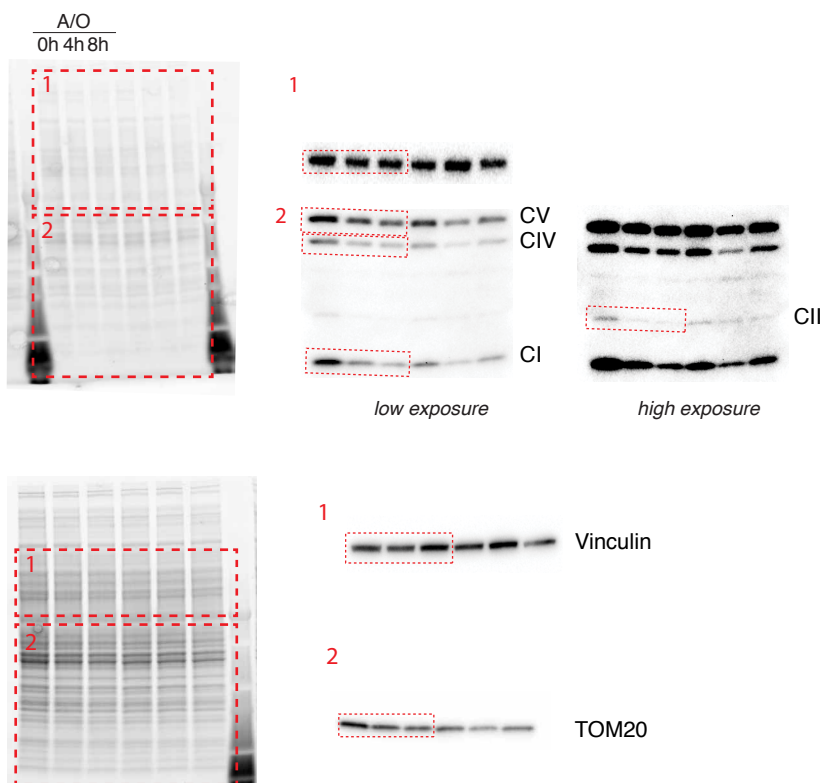

Supplement: Supplementary file 3 — Source Data for Expanded View [file EMBR-22-e50500-s011.zip › EV_Figure_Source_Data/EMBOR-2020-50500V3-FigureEV3_Source_Data-sd/FigureEV3_uncropped gels.pdf]

**FIG EV2A**

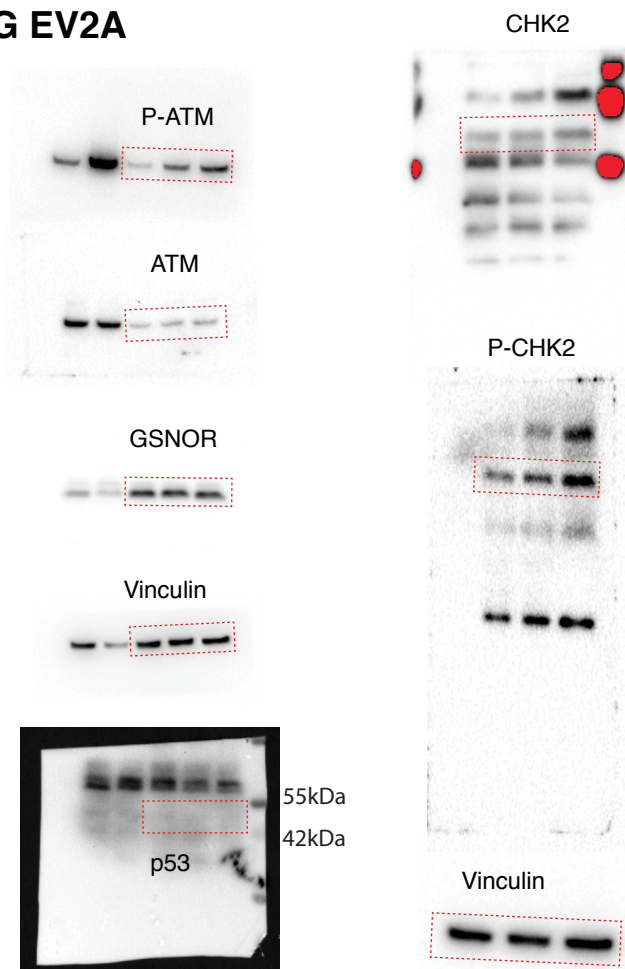

**FIG EV2B**

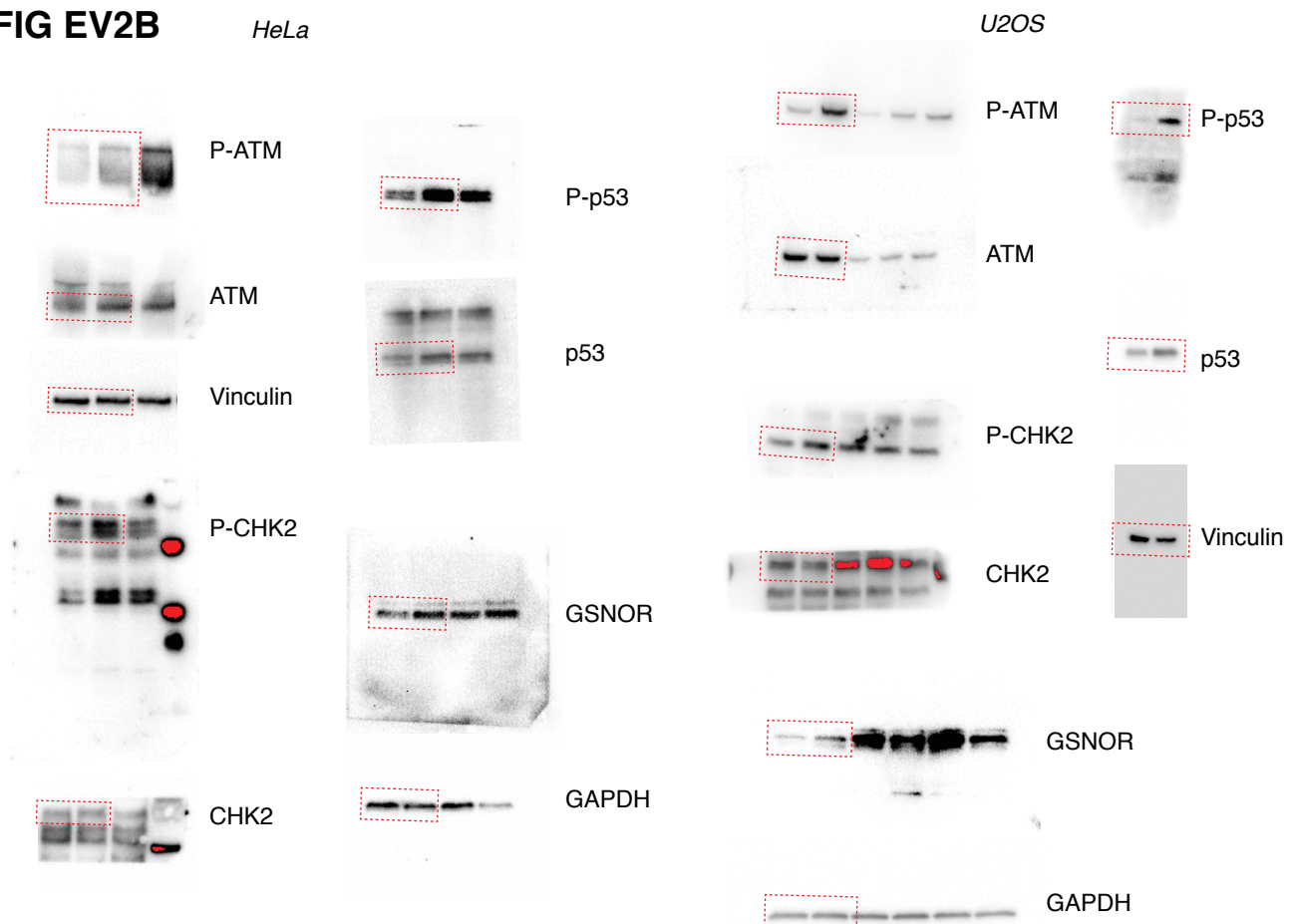

Supplement: Supplementary file 3 — Source Data for Expanded View [file EMBR-22-e50500-s011.zip › EV_Figure_Source_Data/EMBOR-2020-50500V3-FigureEV2_Source_Data-sd/FigureEV2_uncropped gels.pdf]

**FIG 1A**

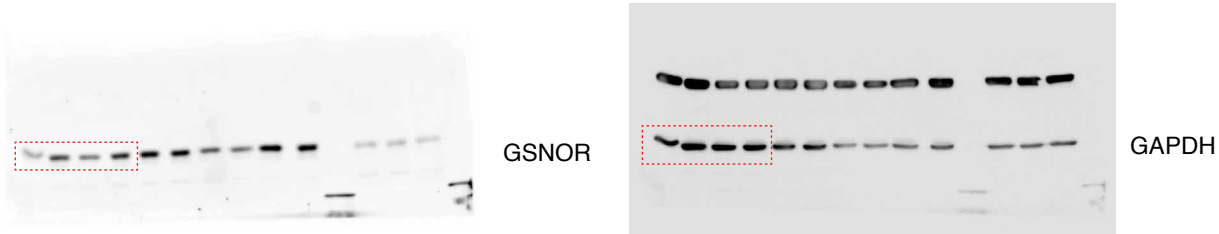

**FIG 1B**

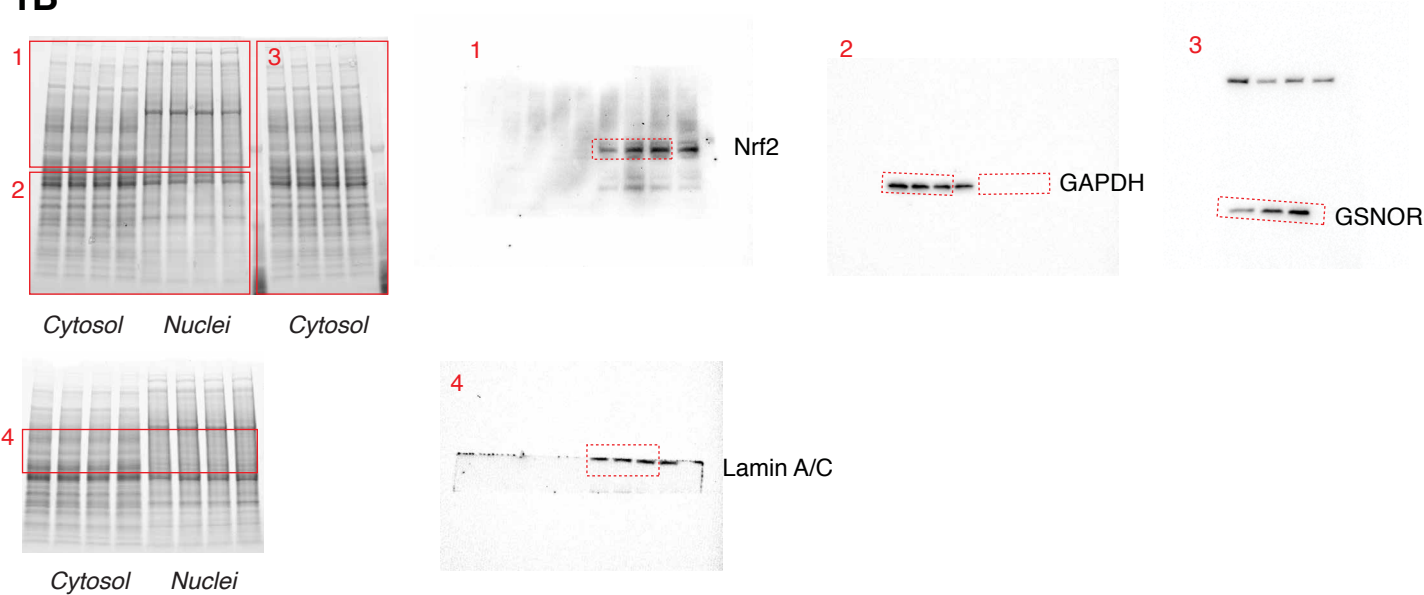

**FIG 1E**

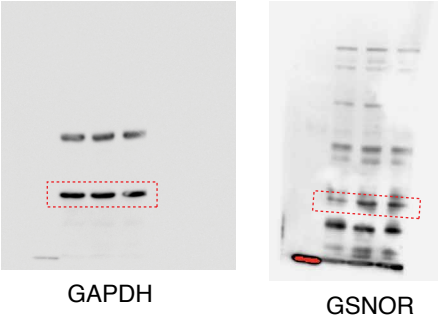

**FIG 1F**

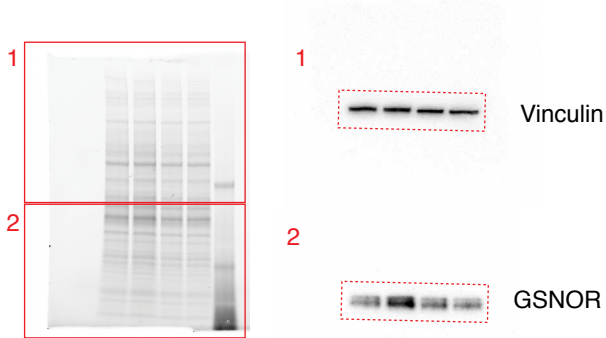

**FIG 1G**

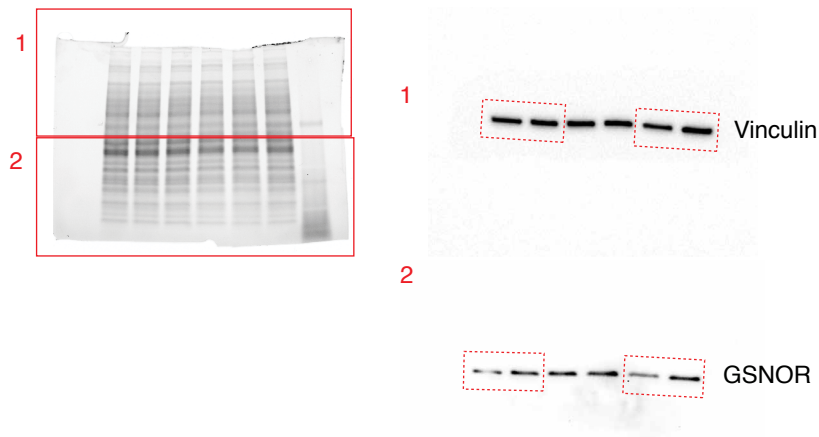

**FIG 1J**

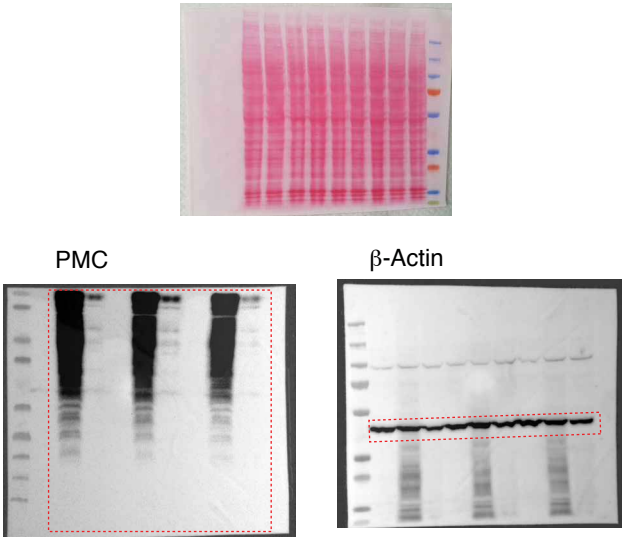

Supplement: Supplementary file 5 — Source Data for Figure 1 [file EMBR-22-e50500-s003.zip › Figure1_uncropped gels.pdf]

FIG 2A

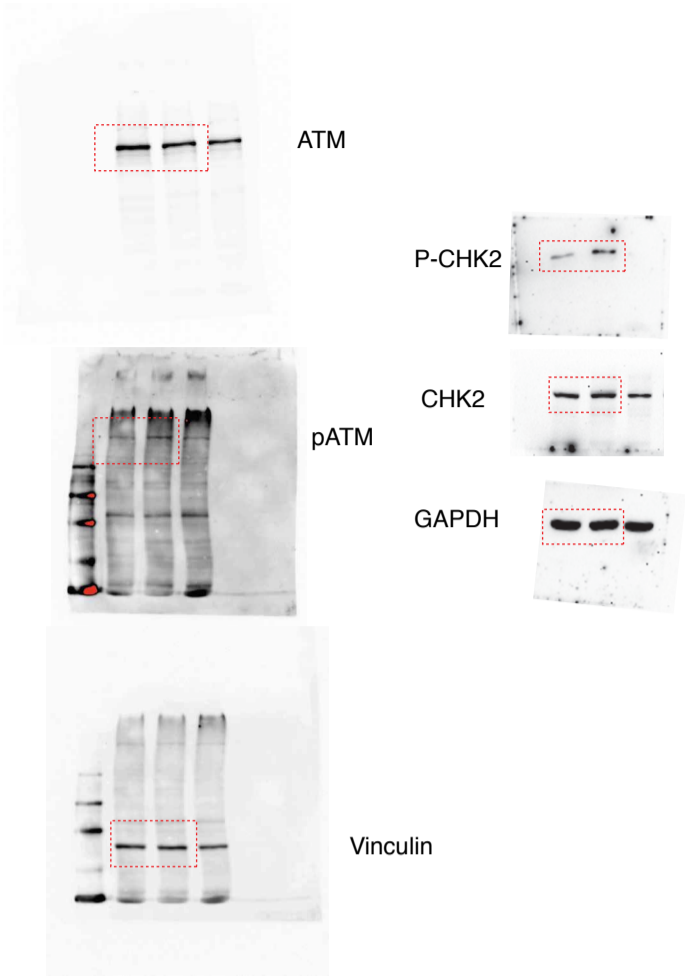

FIG 2B

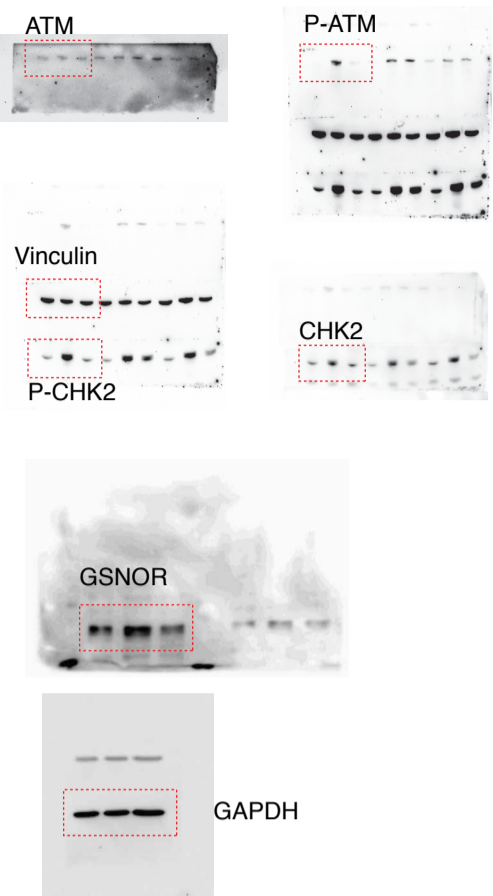

FIG 2C

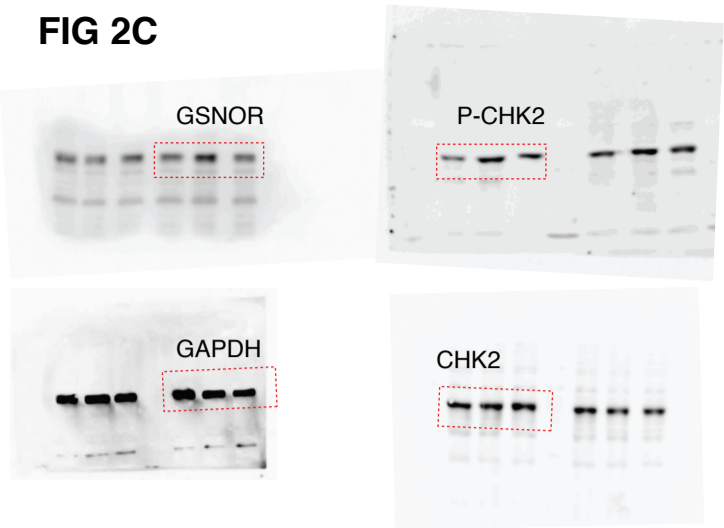

FIG 2D

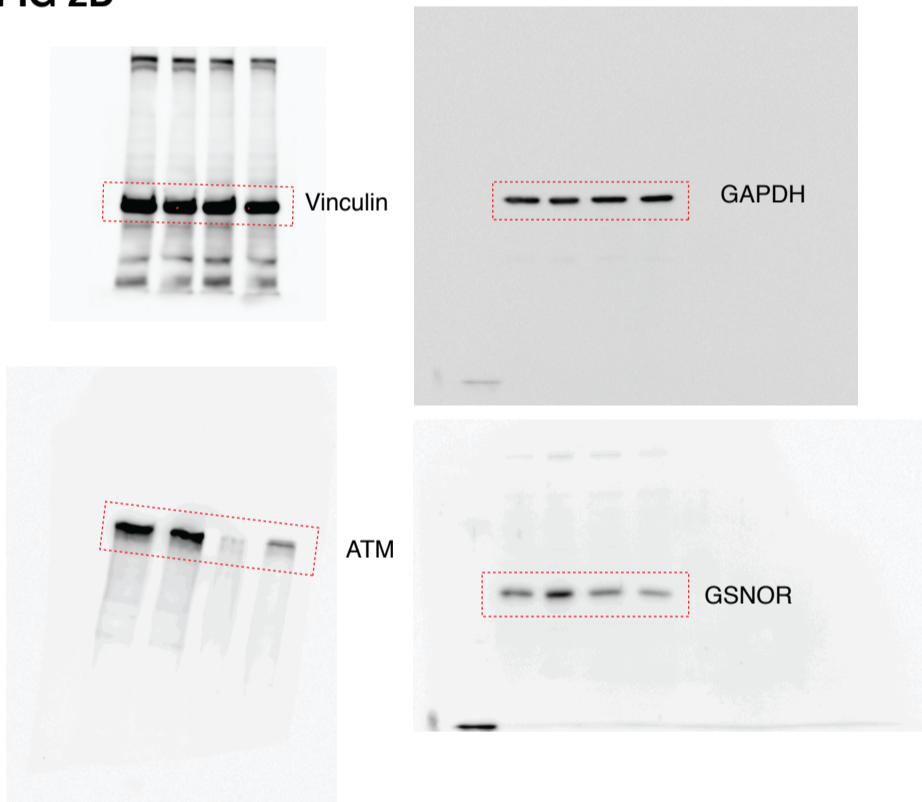

FIG 2E

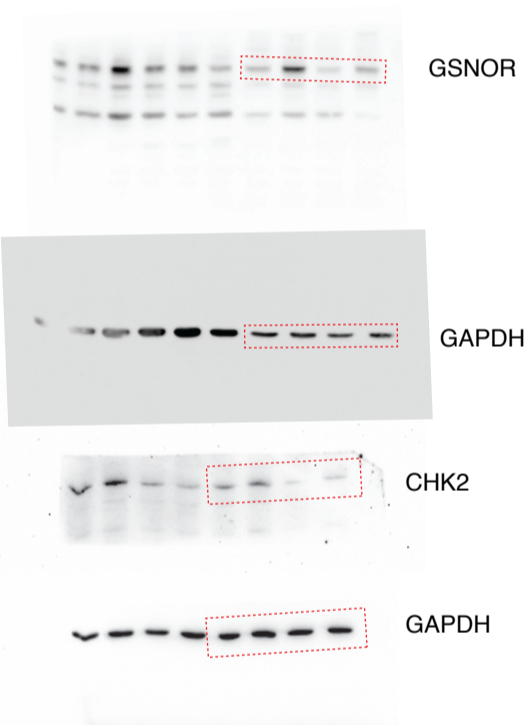

FIG 2F

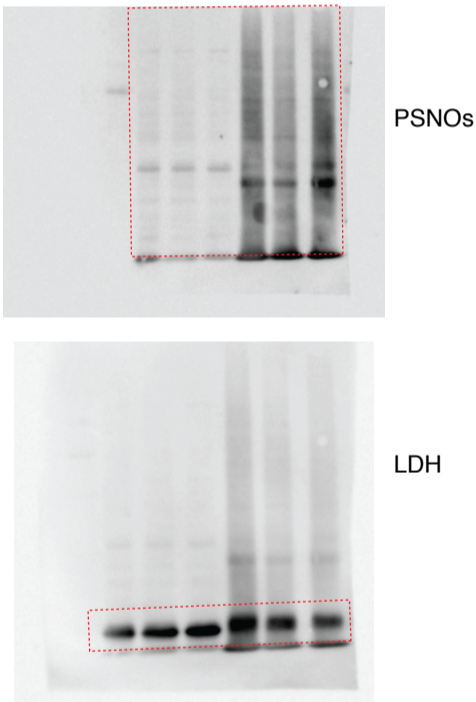

FIG 2H

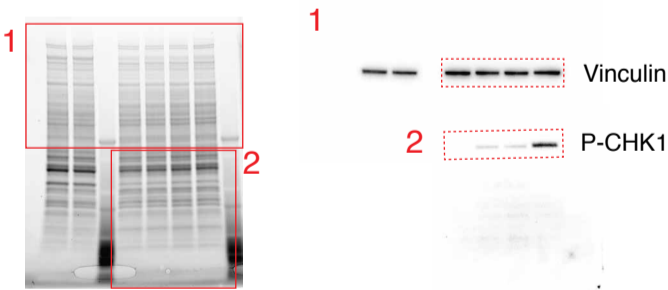

FIG 2I

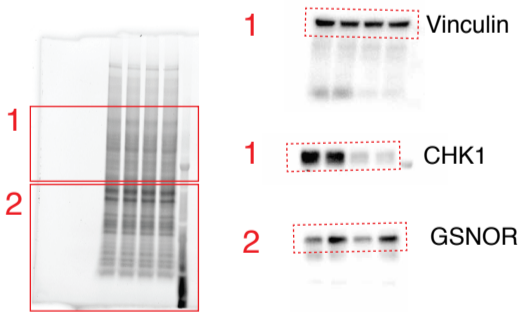

Supplement: Supplementary file 6 — Source Data for Figure 2 [file EMBR-22-e50500-s004.zip › Figure2_uncropped gels.pdf]

**FIG 3B**

GAPDH  $\gamma$ H2AX

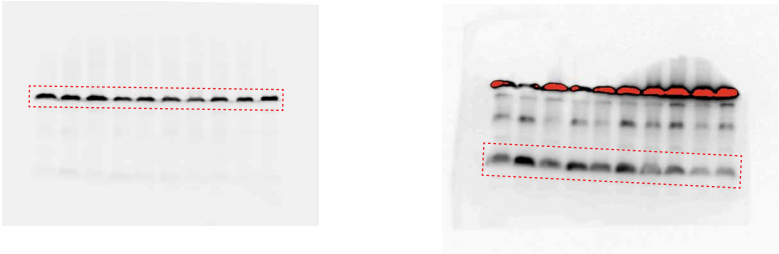

**FIG 3C**

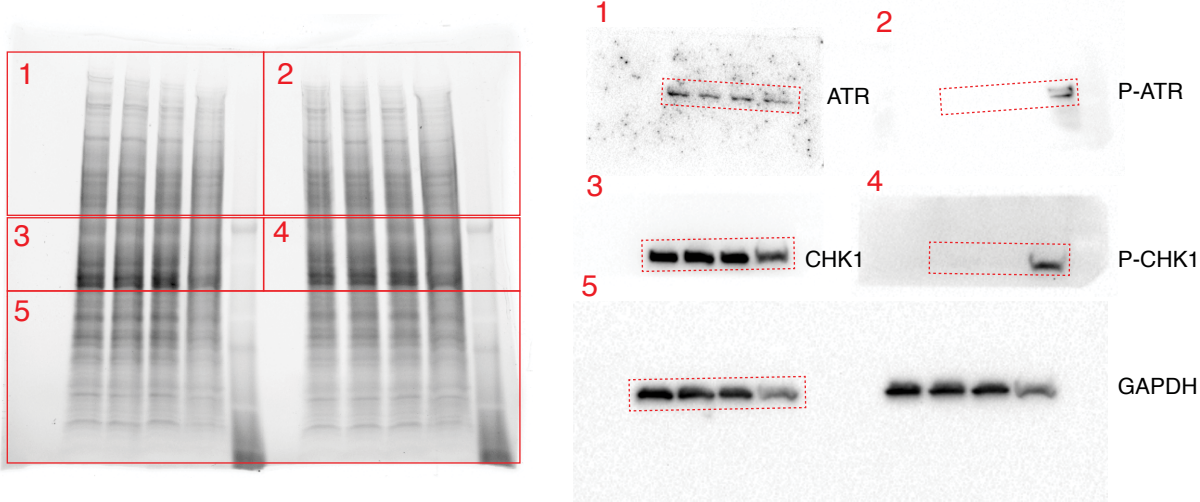

**FIG 3D**

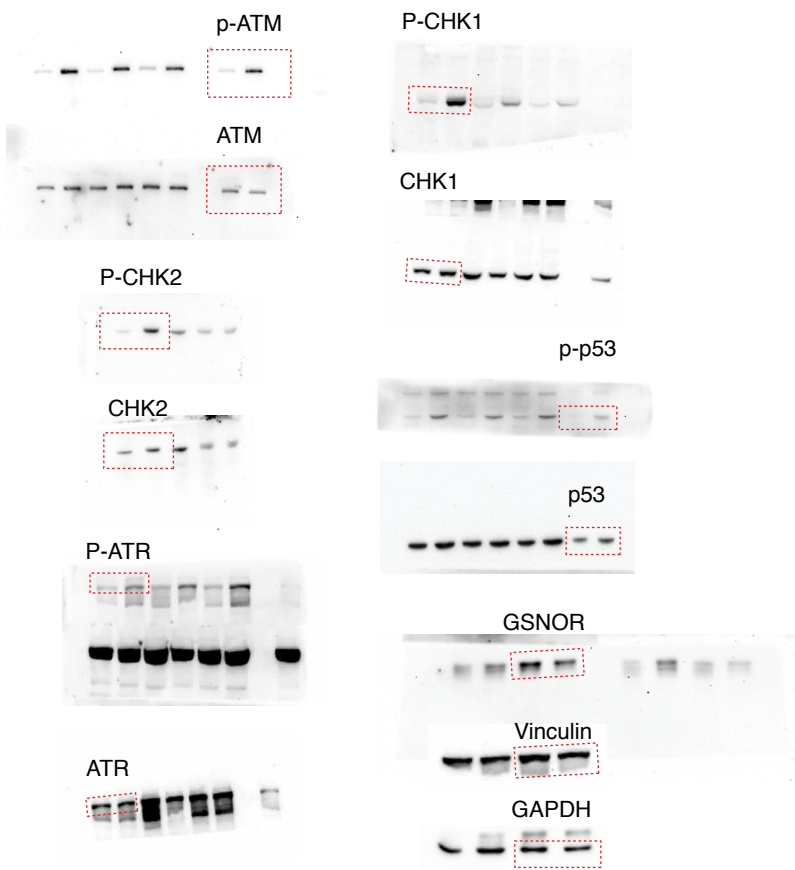

**FIG 3F**

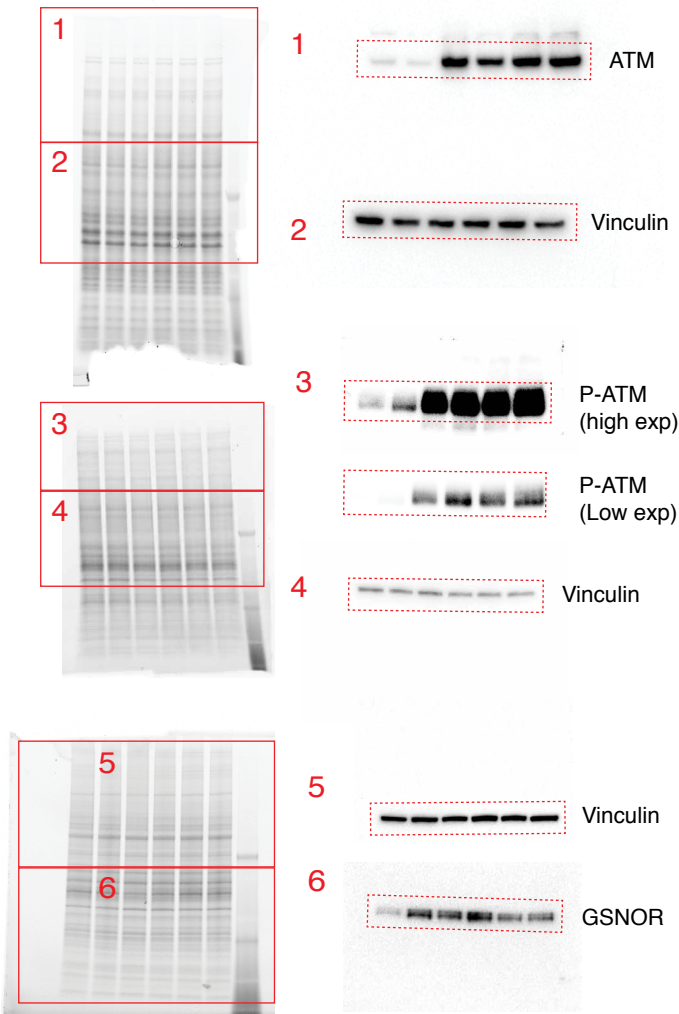

Supplement: Supplementary file 7 — Source Data for Figure 3 [file EMBR-22-e50500-s005.zip › Figure3_uncropped gels.pdf]

**FIG 4A**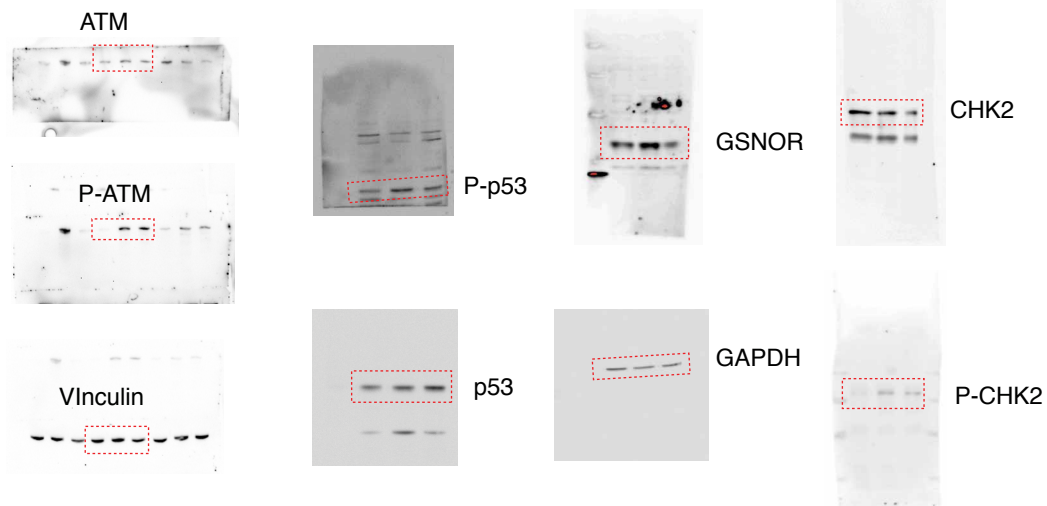**FIG 4B**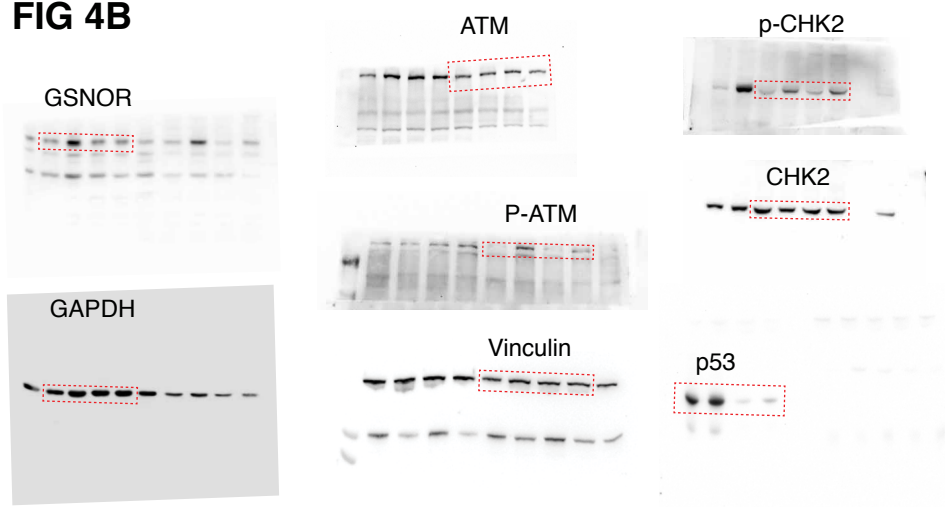**FIG 4C**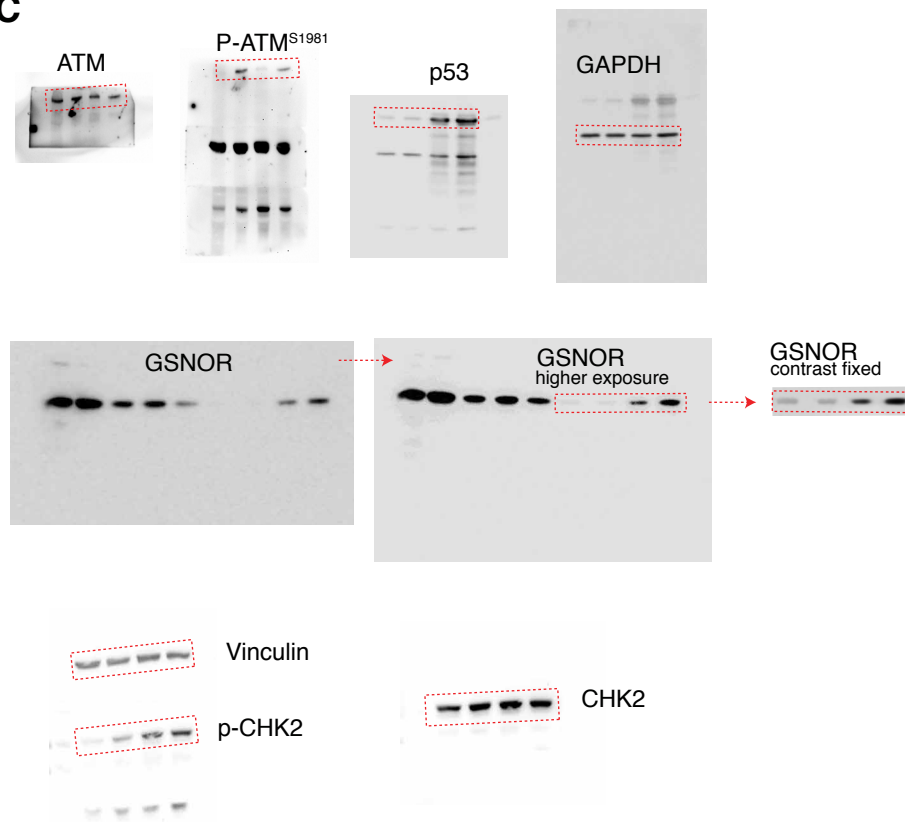

Supplement: Supplementary file 8 — Source Data for Figure 4 [file EMBR-22-e50500-s006.zip › Figure4_uncropped gels.pdf]

FIG 5C

|                               | siSCR |   | siGSNOR |   | siATM |   |
|-------------------------------|-------|---|---------|---|-------|---|
| H <sub>2</sub> O <sub>2</sub> | -     | + | -       | + | -     | + |

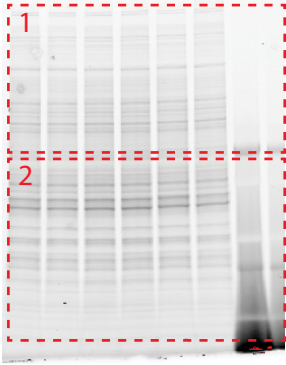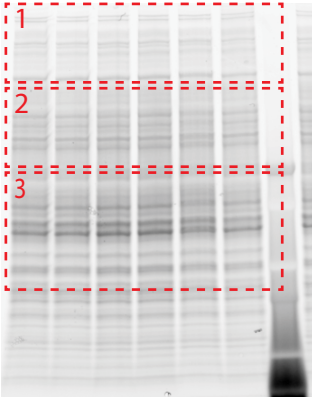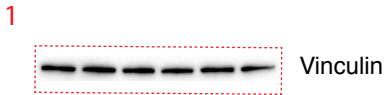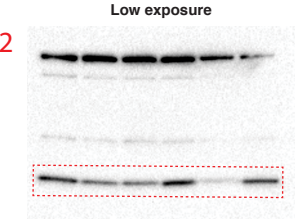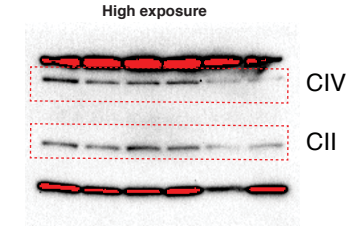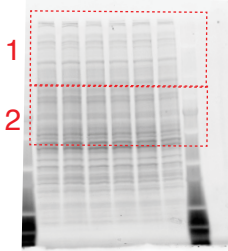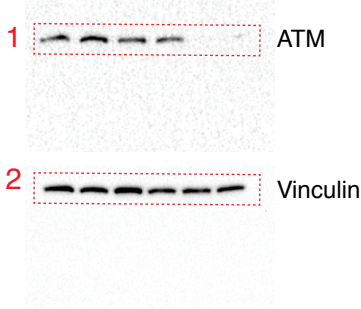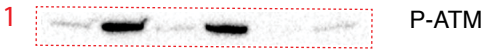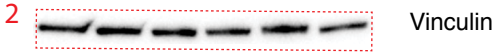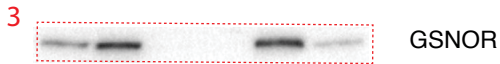

Supplement: Supplementary file 9 — Source Data for Figure 5 [file EMBR-22-e50500-s007.zip › Figure5_uncropped gels.pdf]

FIG 6C

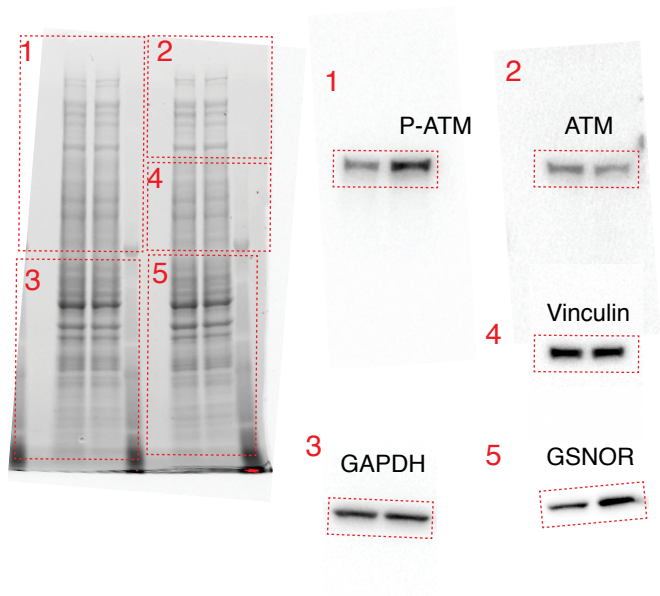

FIG 6G

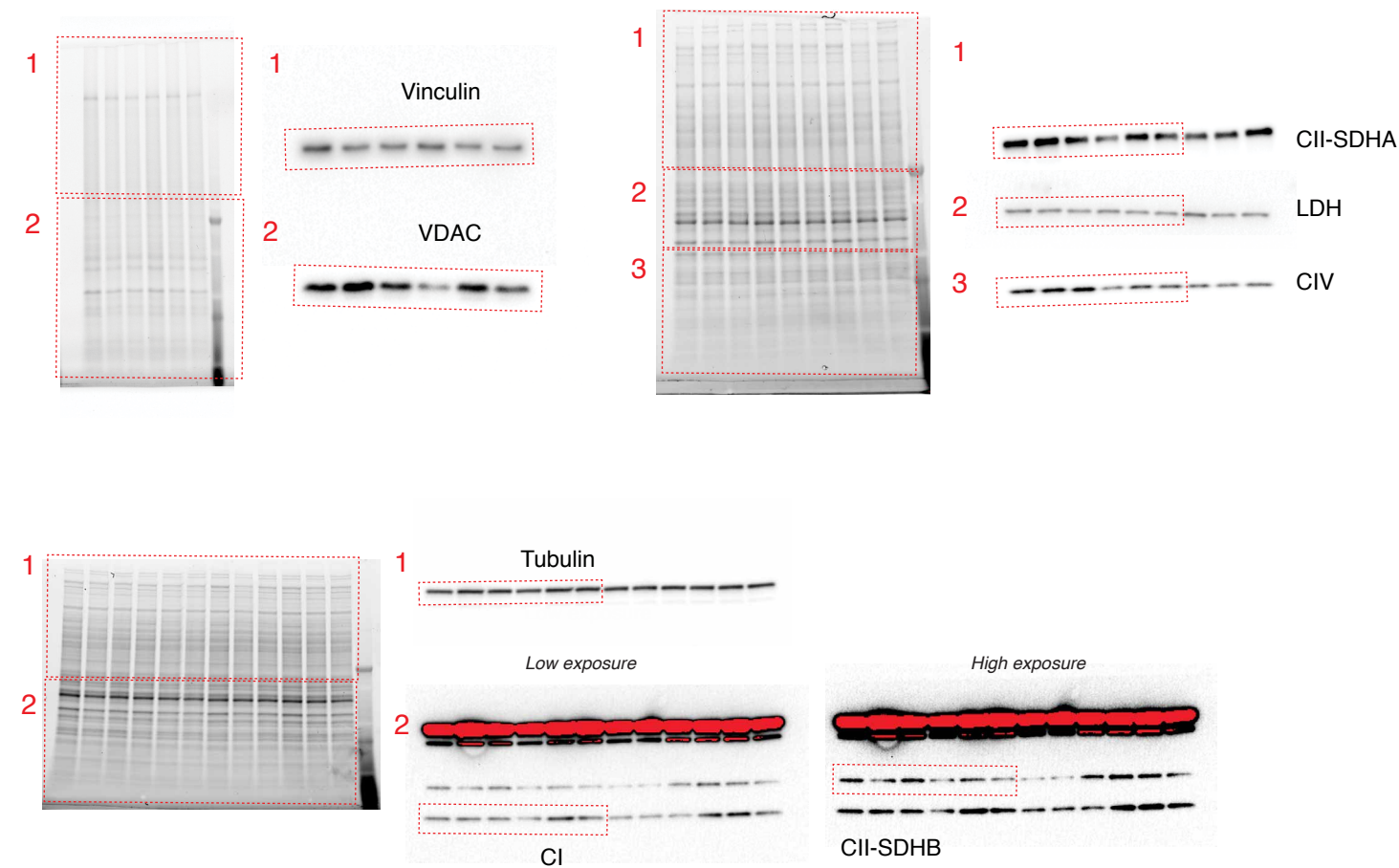

Supplement: Supplementary file 10 — Source Data for Figure 6 [file EMBR-22-e50500-s008.zip › Figure6_uncropped gels.pdf]

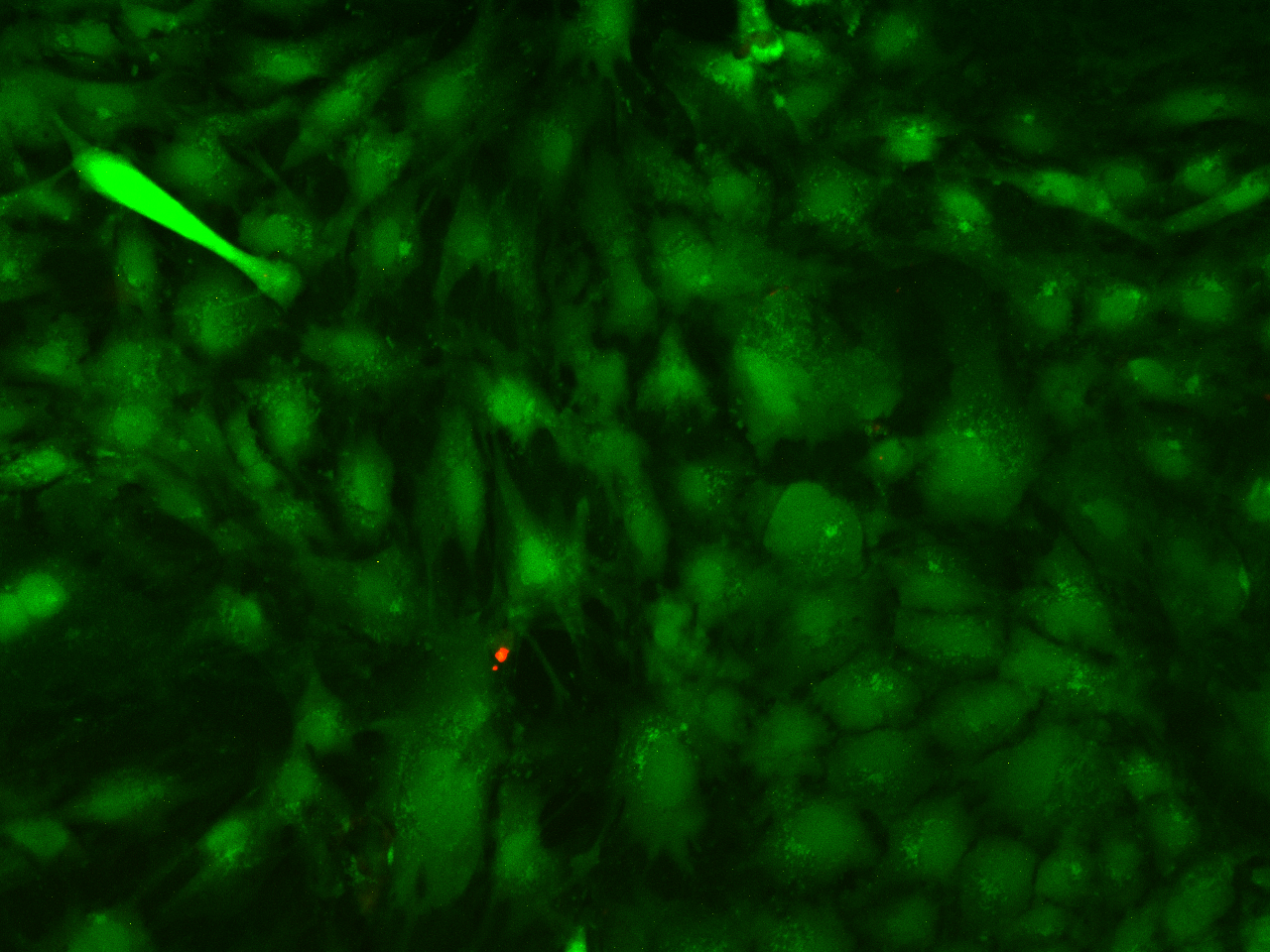

Supplement: Supplementary file 11 — Source Data for Figure 7 [file EMBR-22-e50500-s009.zip › Fig7H_source images/WT, DPTA merge.png]

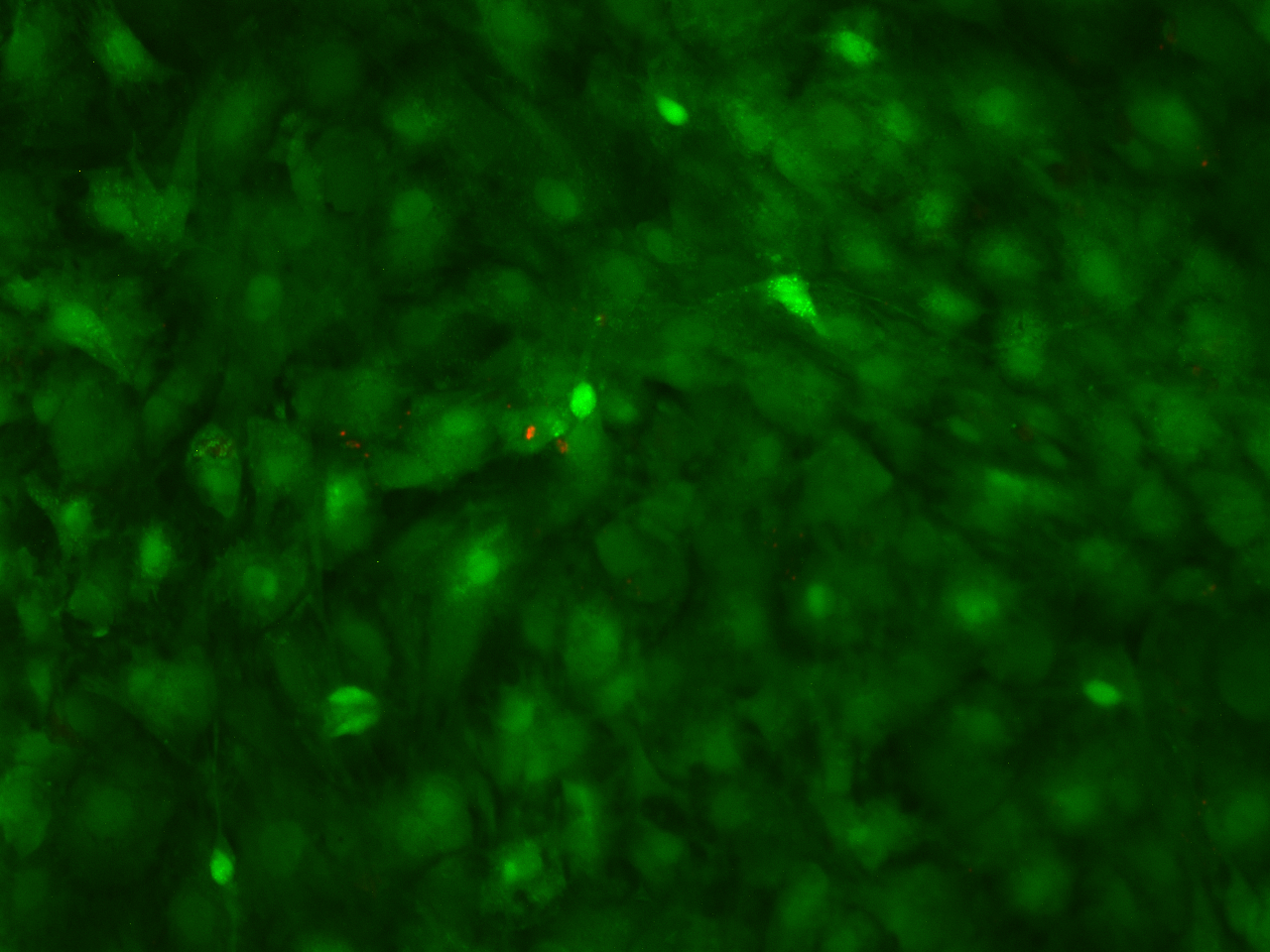

Supplement: Supplementary file 11 — Source Data for Figure 7 [file EMBR-22-e50500-s009.zip › Fig7H_source images/KO, CTR merge.png]

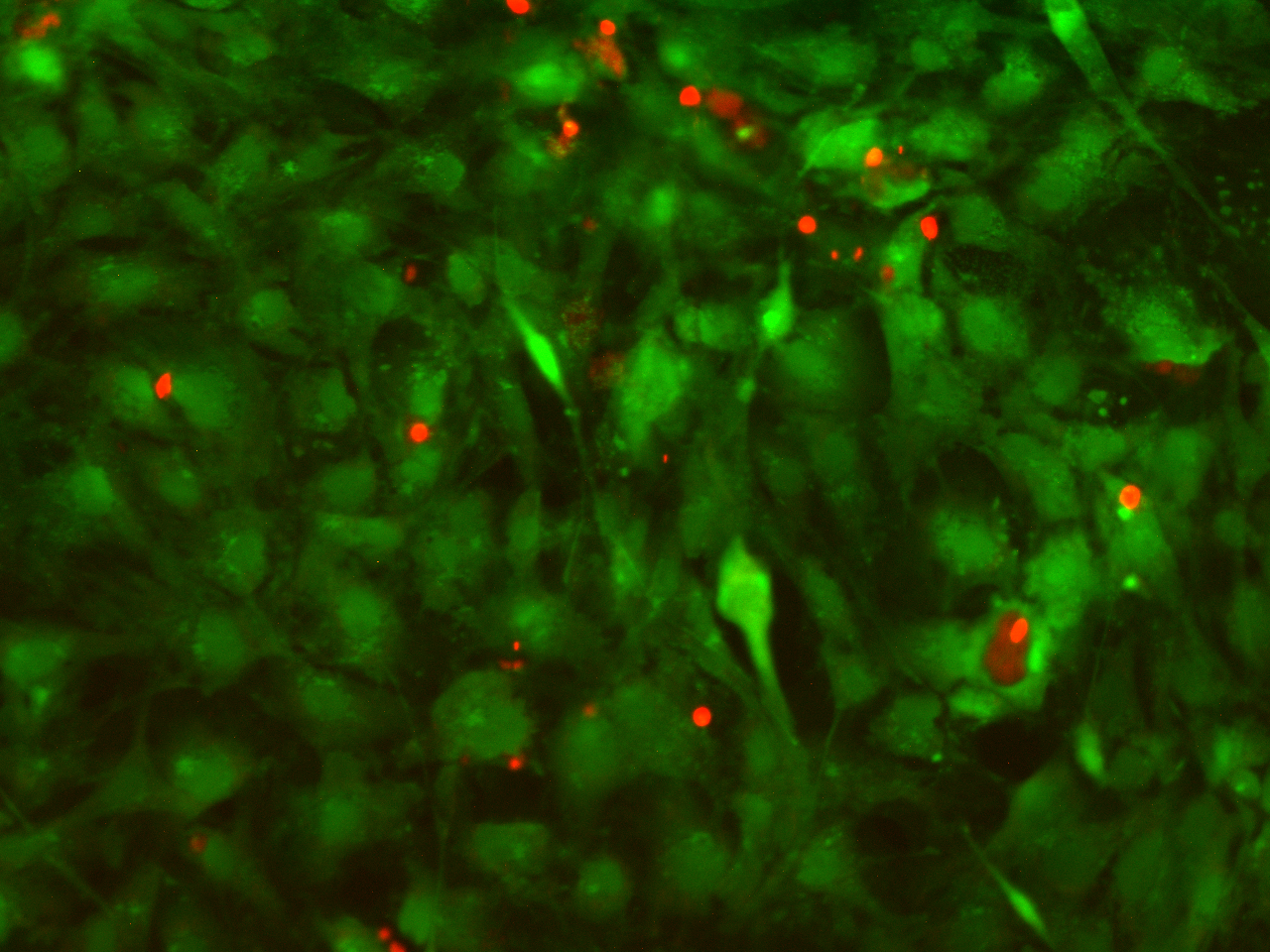

Supplement: Supplementary file 11 — Source Data for Figure 7 [file EMBR-22-e50500-s009.zip › Fig7H_source images/KO, H2O2-DPTA merge.png]

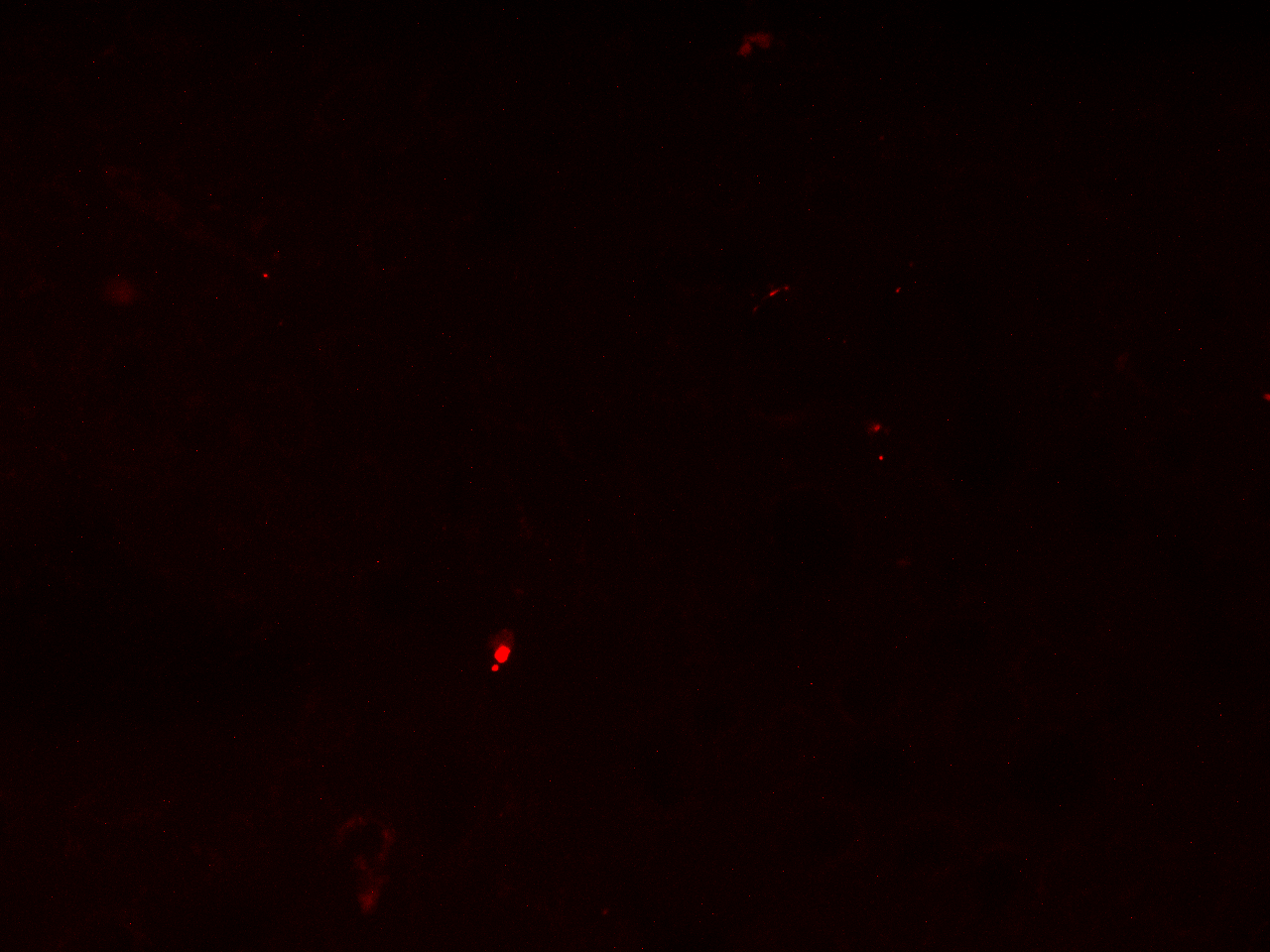

Supplement: Supplementary file 11 — Source Data for Figure 7 [file EMBR-22-e50500-s009.zip › Fig7H_source images/WT, DPTA red.png]

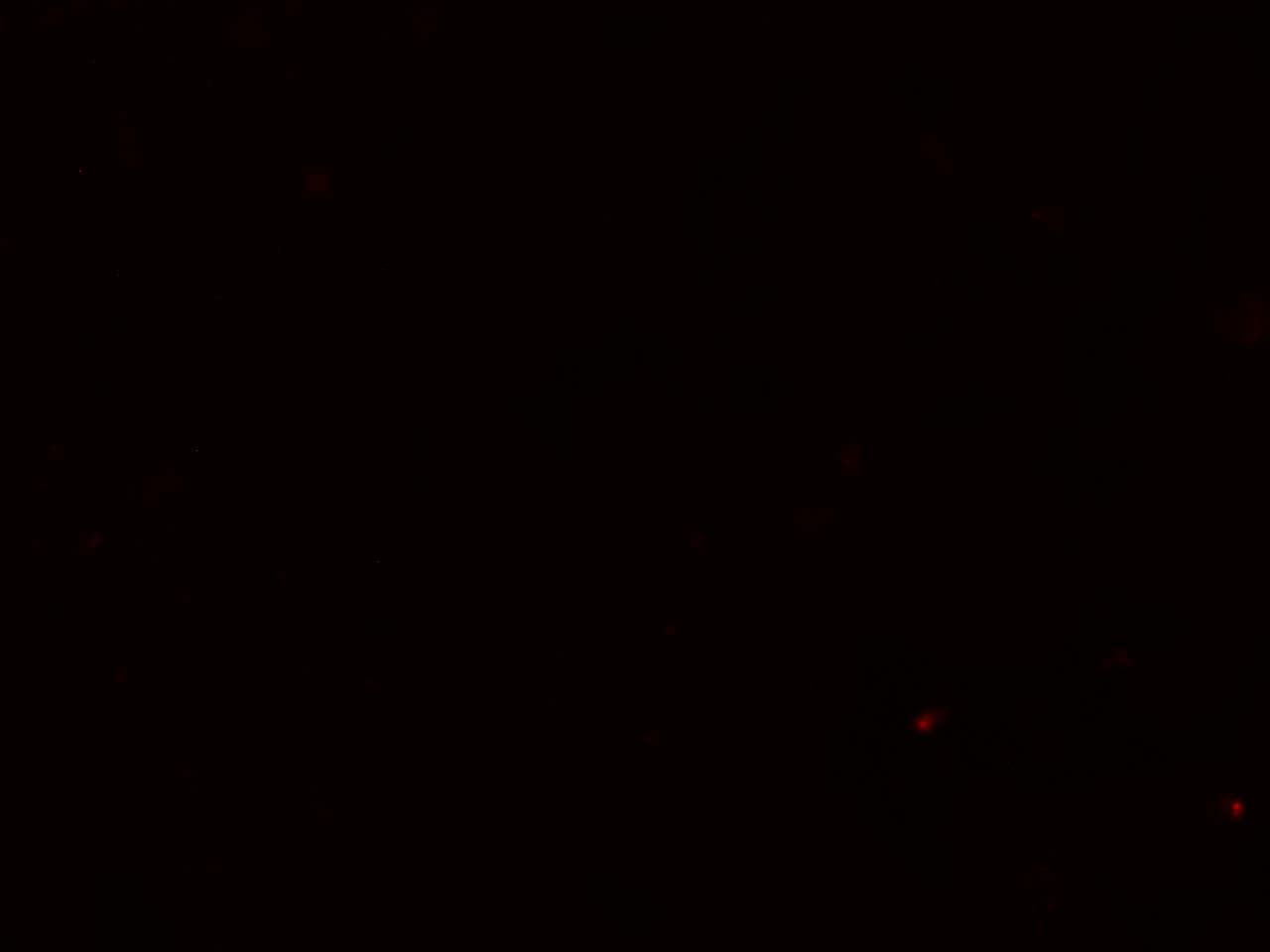

Supplement: Supplementary file 11 — Source Data for Figure 7 [file EMBR-22-e50500-s009.zip › Fig7H_source images/WT, CTR red.png]

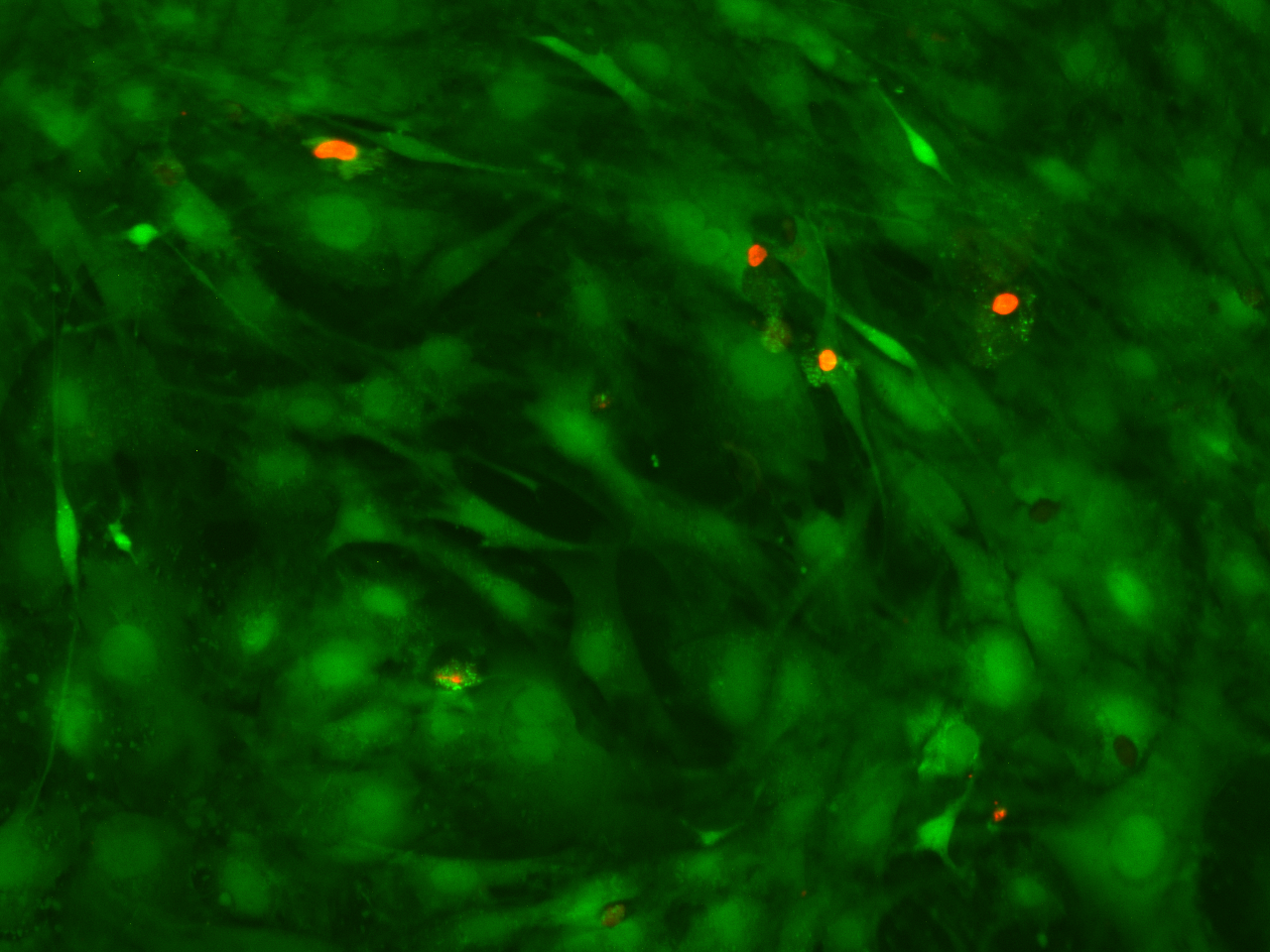

Supplement: Supplementary file 11 — Source Data for Figure 7 [file EMBR-22-e50500-s009.zip › Fig7H_source images/KO, H2O2 merge.png]

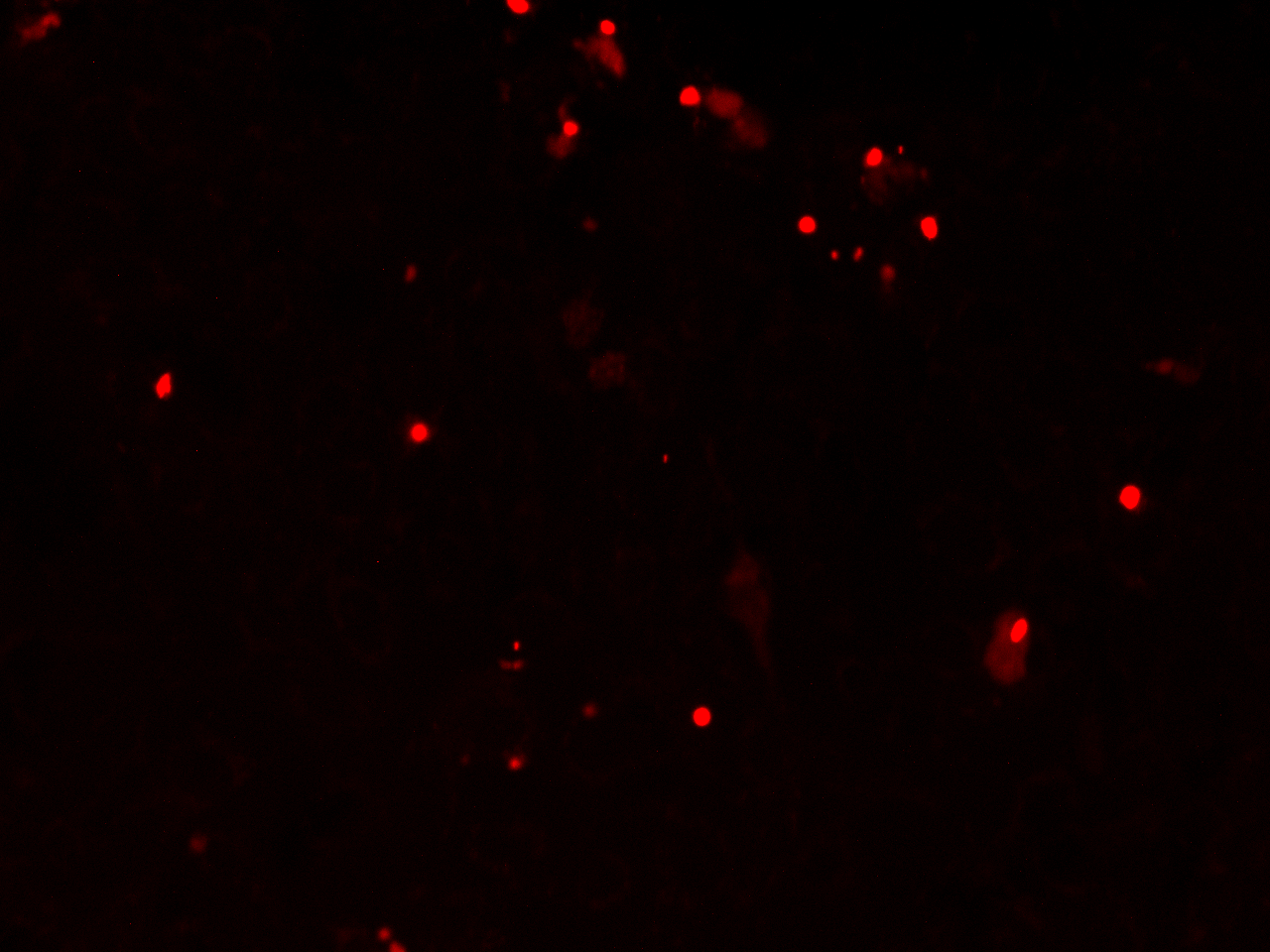

Supplement: Supplementary file 11 — Source Data for Figure 7 [file EMBR-22-e50500-s009.zip › Fig7H_source images/KO, H2O2-DPTA red.png]

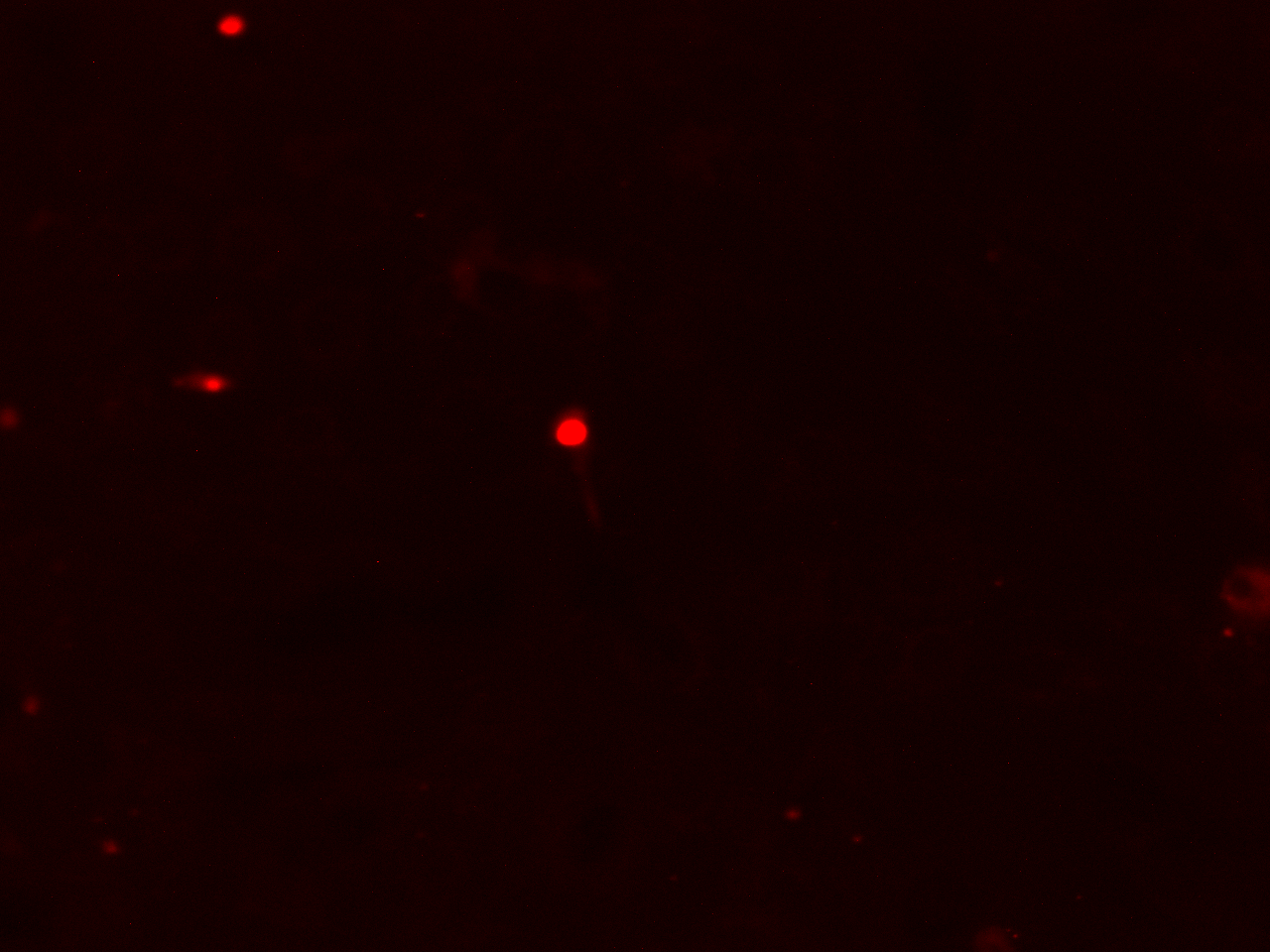

Supplement: Supplementary file 11 — Source Data for Figure 7 [file EMBR-22-e50500-s009.zip › Fig7H_source images/WT, H2O2 red.png]

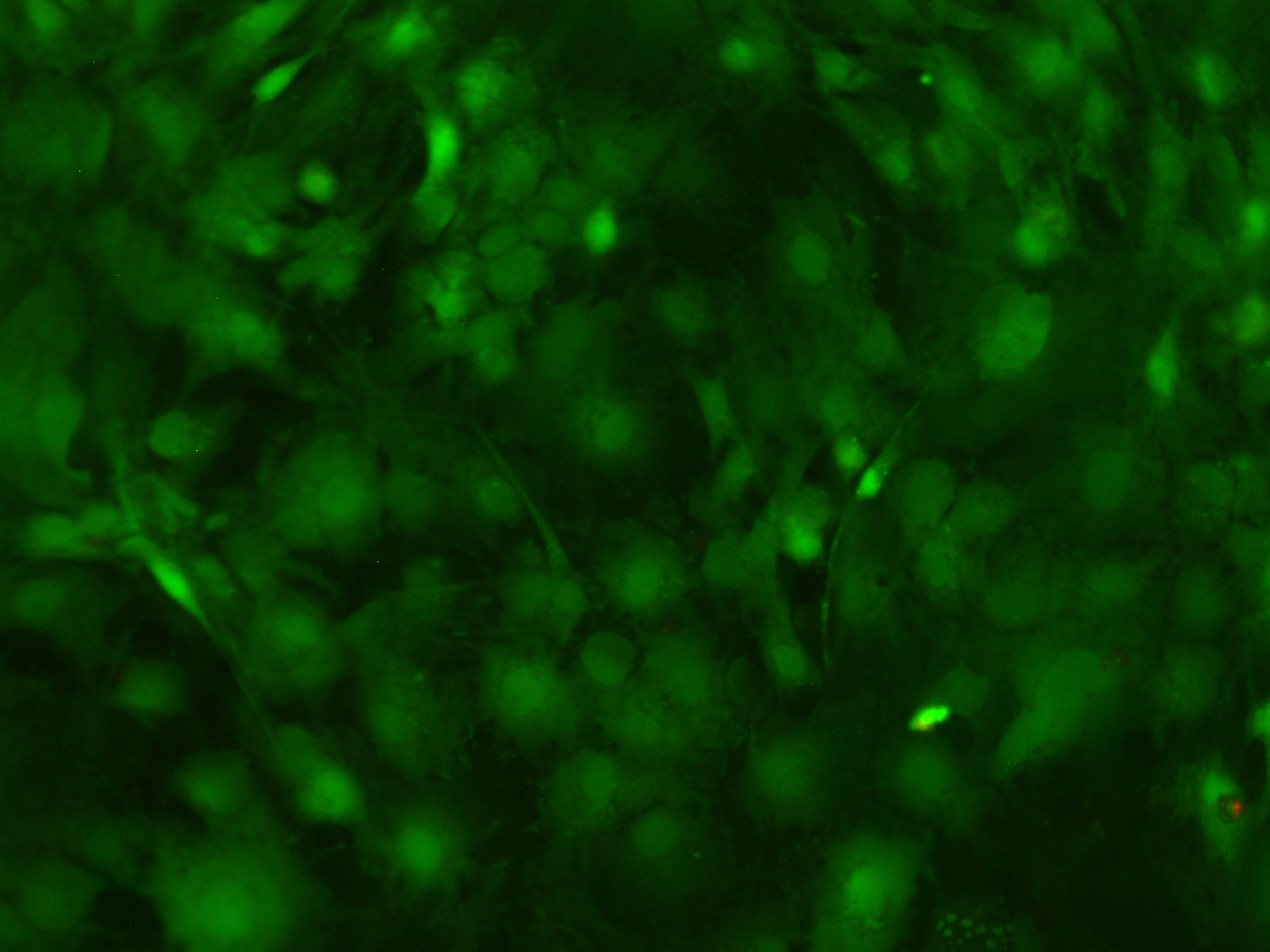

Supplement: Supplementary file 11 — Source Data for Figure 7 [file EMBR-22-e50500-s009.zip › Fig7H_source images/WT, CTR merge.png]

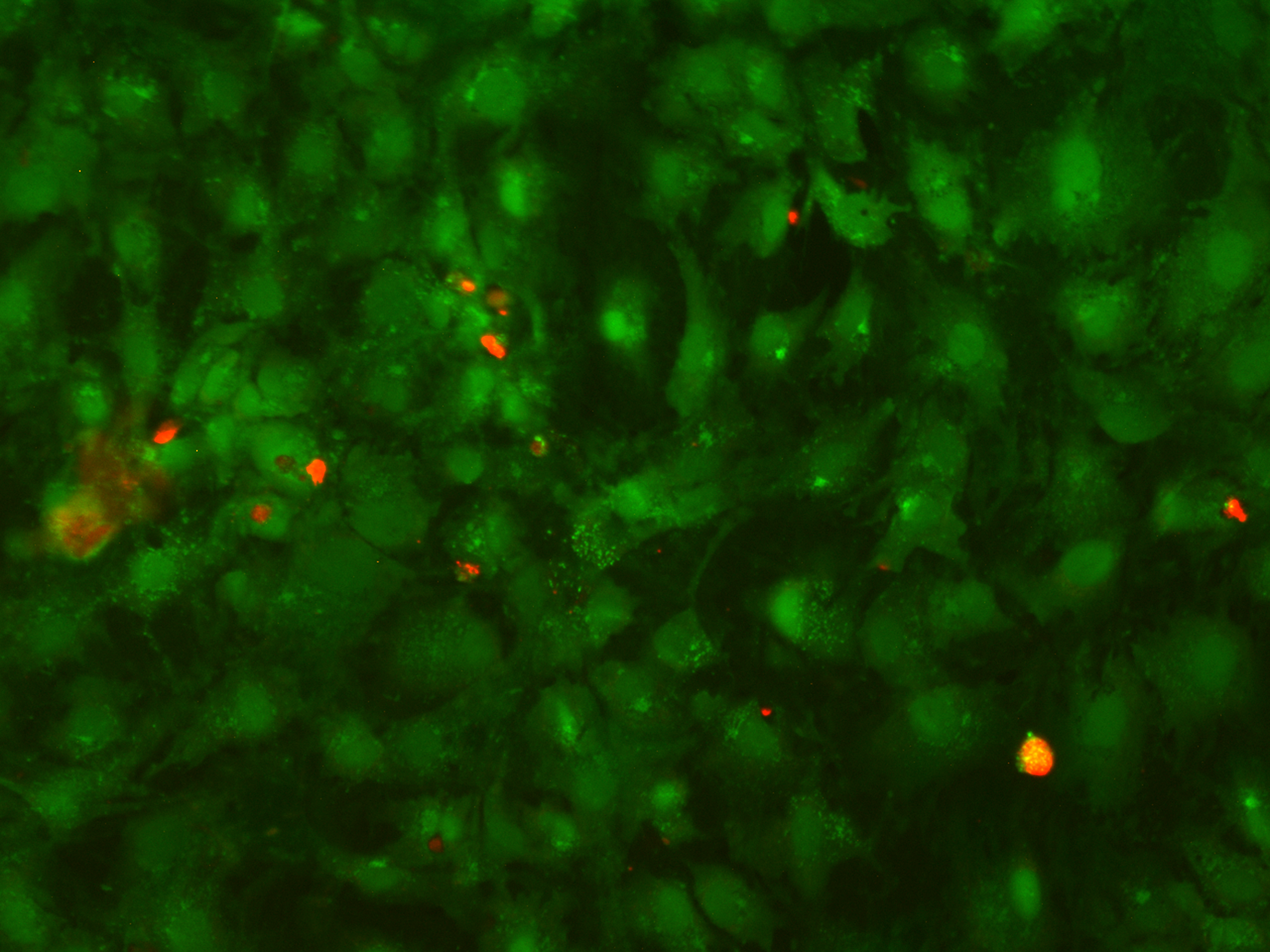

Supplement: Supplementary file 11 — Source Data for Figure 7 [file EMBR-22-e50500-s009.zip › Fig7H_source images/KO, DPTA merge.png]

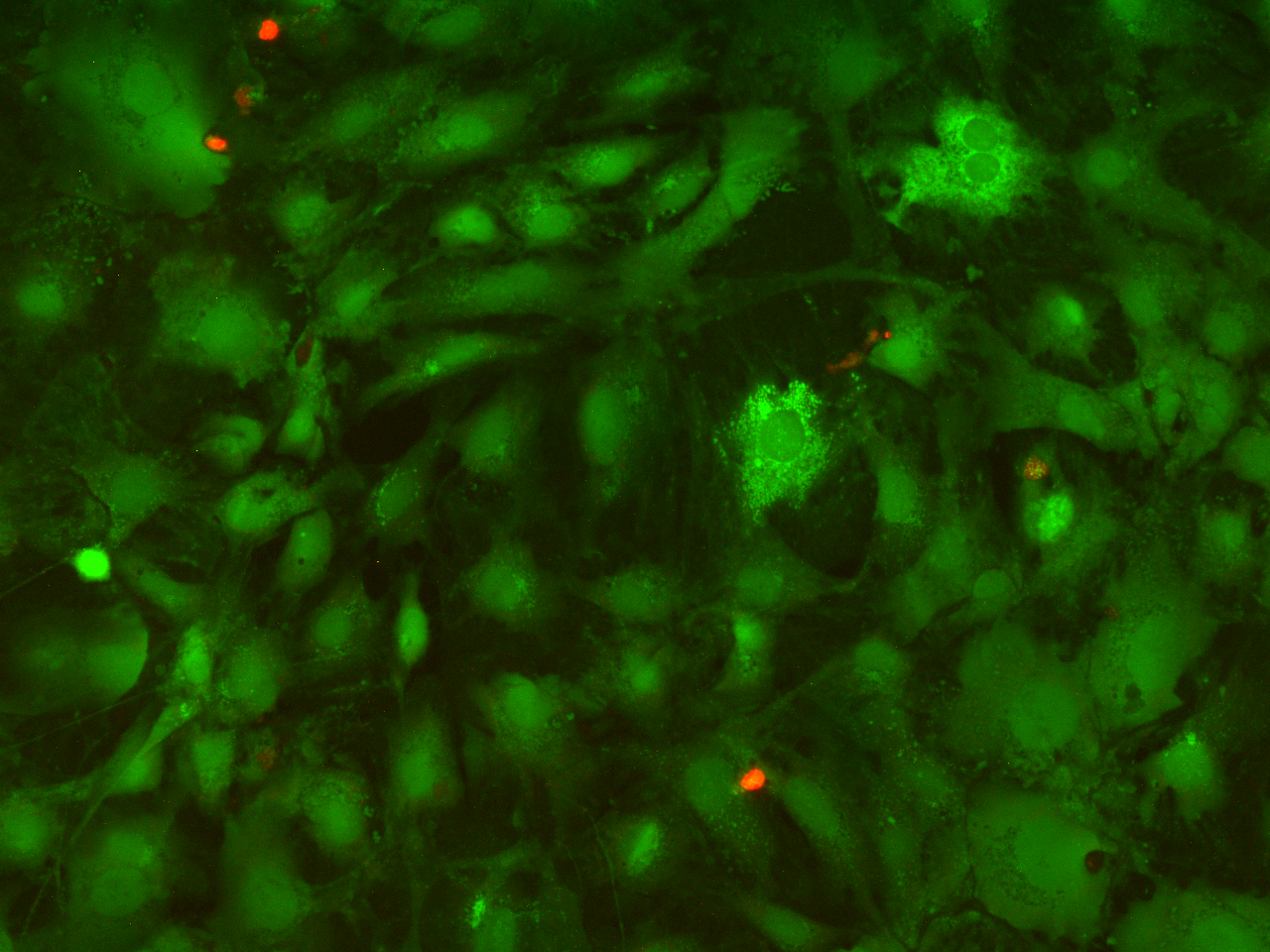

Supplement: Supplementary file 11 — Source Data for Figure 7 [file EMBR-22-e50500-s009.zip › Fig7H_source images/WT, H2O2-DPTA merge.png]

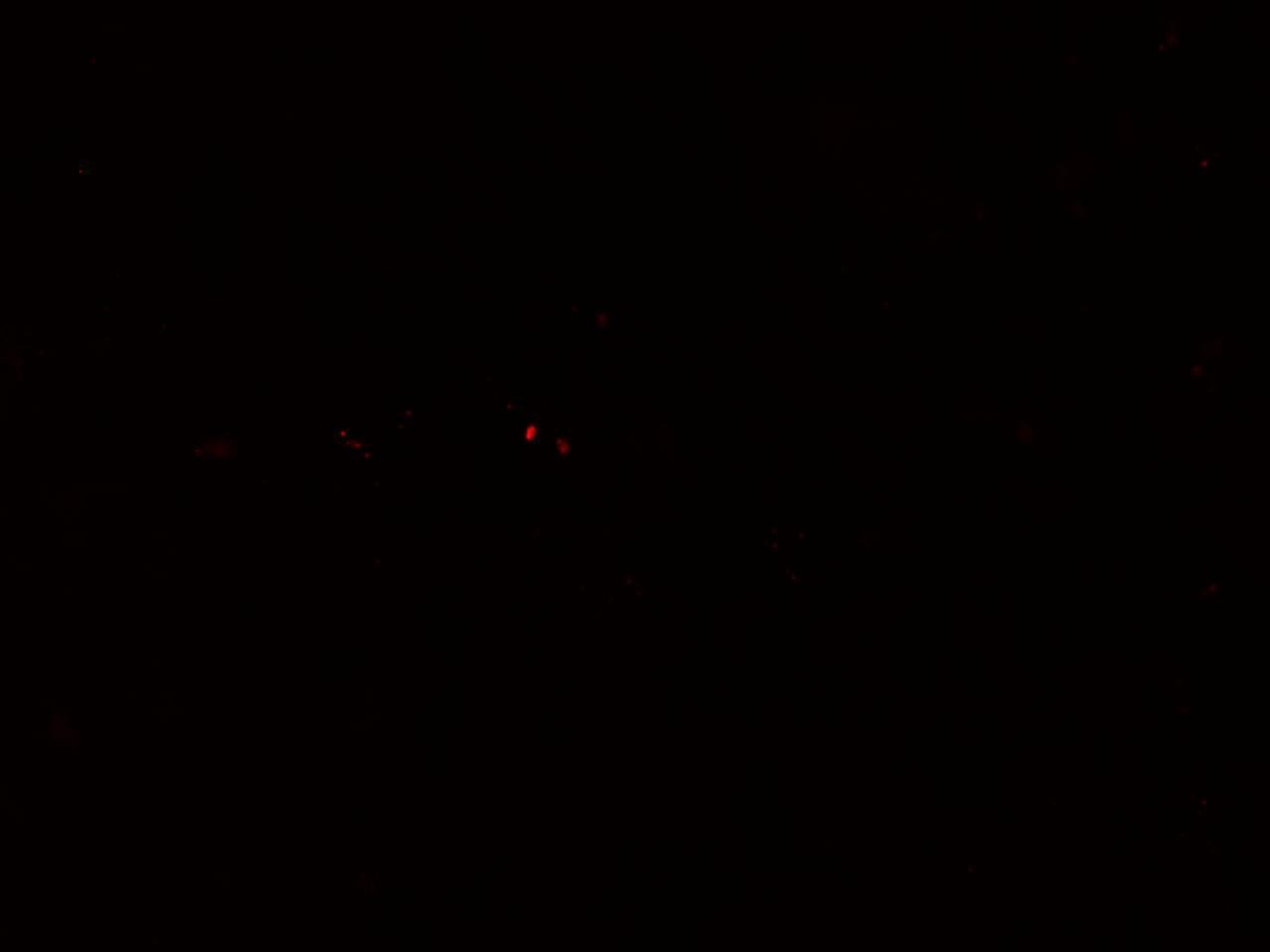

Supplement: Supplementary file 11 — Source Data for Figure 7 [file EMBR-22-e50500-s009.zip › Fig7H_source images/KO, CTR red.png]

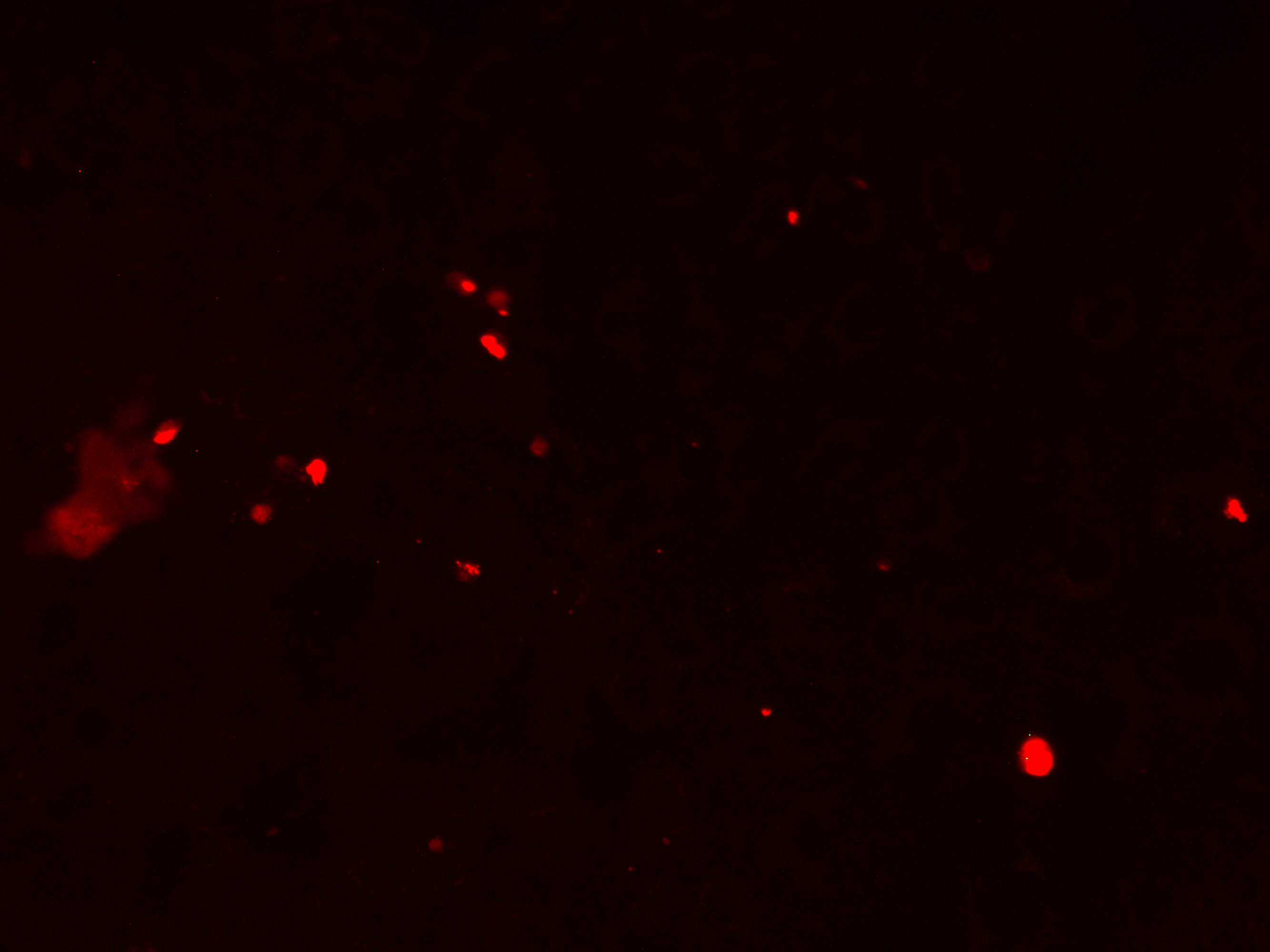

Supplement: Supplementary file 11 — Source Data for Figure 7 [file EMBR-22-e50500-s009.zip › Fig7H_source images/KO, DPTA red.png]

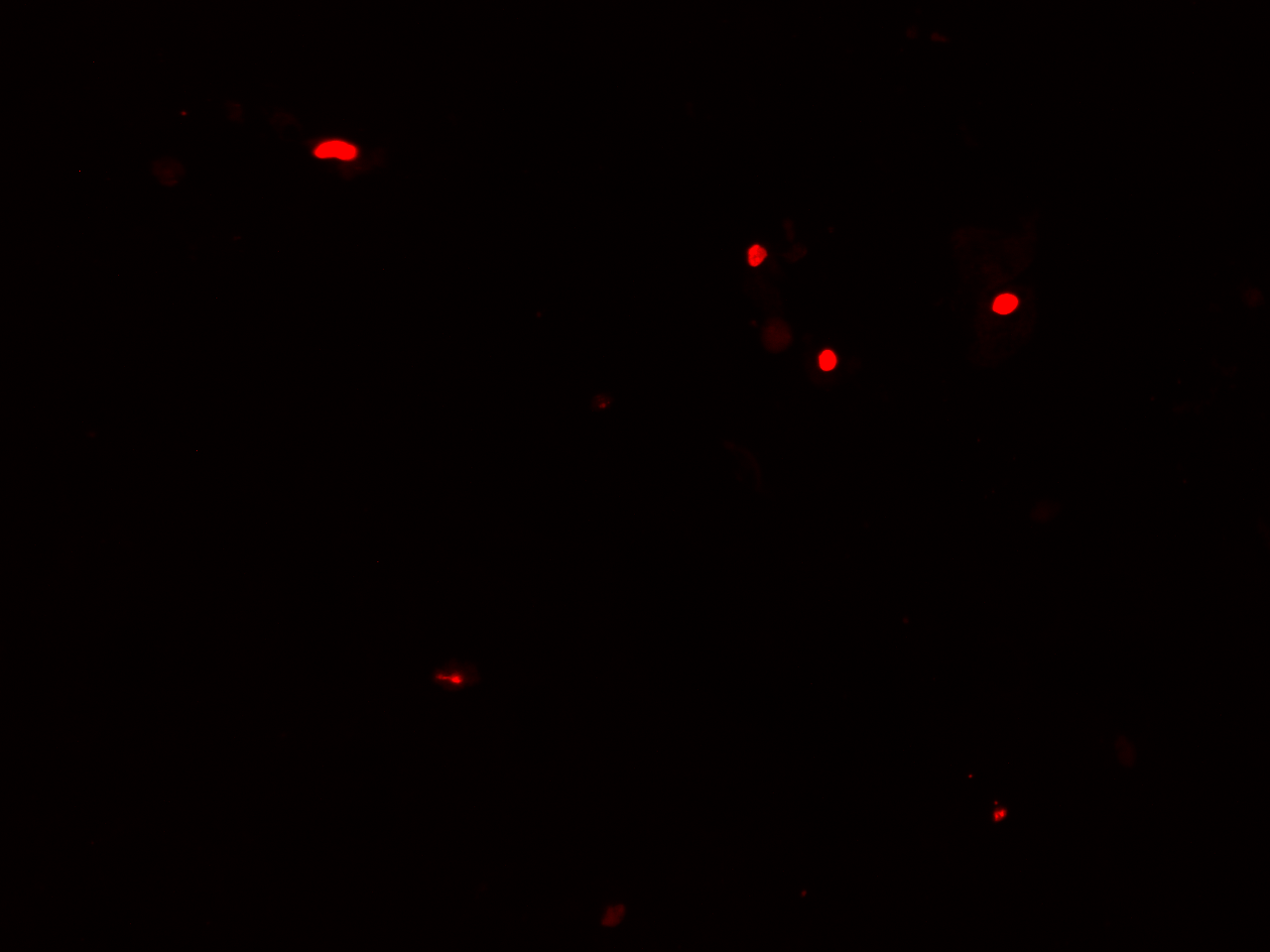

Supplement: Supplementary file 11 — Source Data for Figure 7 [file EMBR-22-e50500-s009.zip › Fig7H_source images/KO, H2O2 red.png]

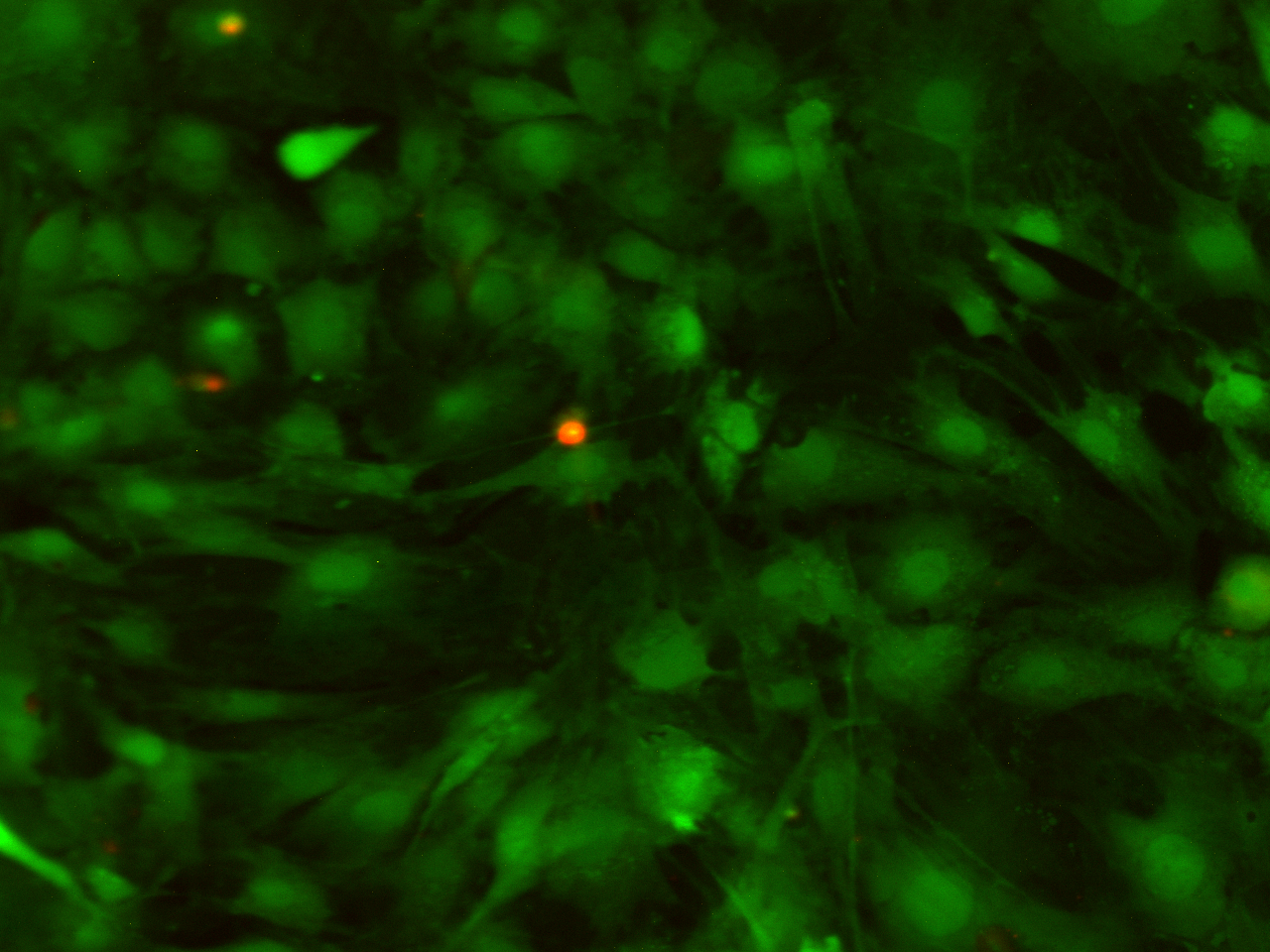

Supplement: Supplementary file 11 — Source Data for Figure 7 [file EMBR-22-e50500-s009.zip › Fig7H_source images/WT, H2O2 merge.png]

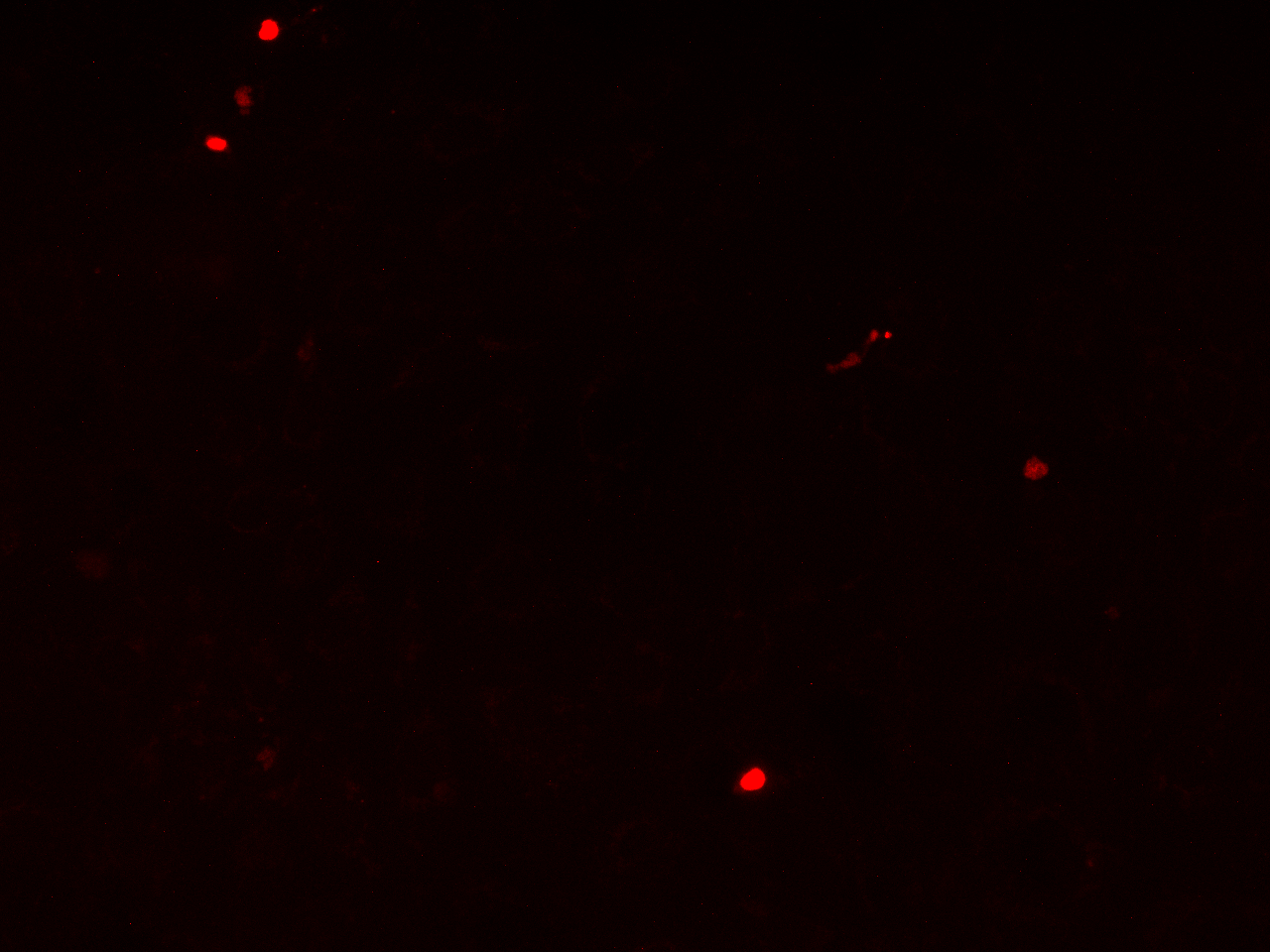

Supplement: Supplementary file 11 — Source Data for Figure 7 [file EMBR-22-e50500-s009.zip › Fig7H_source images/WT, H2O2-DPTA red.png]

**FIG 7B**

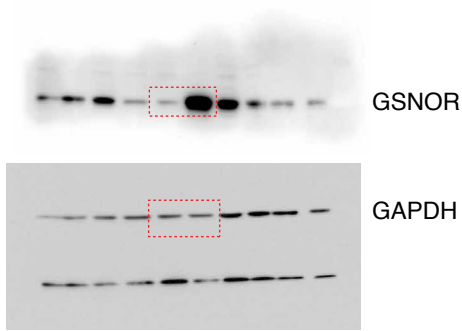

**FIG 7D**

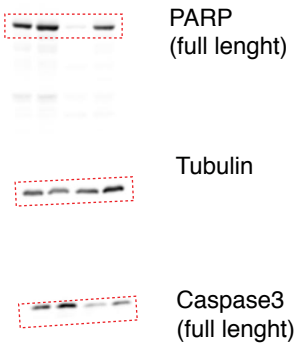

**FIG 7F**

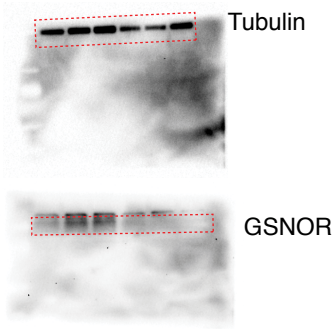

Supplement: Supplementary file 11 — Source Data for Figure 7 [file EMBR-22-e50500-s009.zip › Figure7_uncropped gels.pdf]

**FIG 8C**

Vinculin

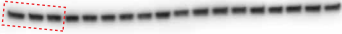

GSNOR

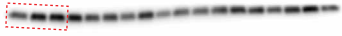

P-ATM

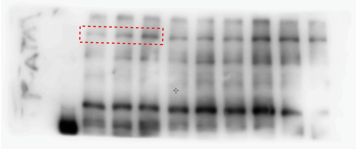

ATM

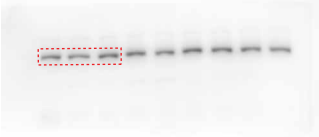

Supplement: Supplementary file 12 — Source Data for Figure 8 [file EMBR-22-e50500-s010.zip › Figure8_uncropped gels.pdf]
